# High-salt diet decreases FOLFOX efficacy via gut bacterial tryptophan metabolism in colorectal cancer

**DOI:** 10.1186/s10020-025-01122-8

**Published:** 2025-02-19

**Authors:** Yufei Deng, Xiaoying Hou, Qian Fang, Haiping Wang, Xiaoxuan Li, Zhiyong Hu, Zhaolu Liu, Limei Fan, Yunyi Liu, Zhengqi Fu, Xiji Shu, Binlian Sun, Lijun Huang, Yuchen Liu

**Affiliations:** 1 Wuhan Institute of Biomedical Sciences, School of Medicine, Jianghan University, Wuhan, 430056 China; 2 Cancer Institute, School of Medicine, Jianghan University, Wuhan, 430056 China; 3 Hubei Key Laboratory of Cognitive and Affective Disorders, Jianghan University, Wuhan, 430056 China; 4 Department of Pathology, Renmin Hospital of Huangpi District of Jianghan University, Wuhan, 430399 China; 5 Department of Gastrointestinal Surgery, The Third Affiliated Hospital of Sun Yat-sen University, Guangzhou, 510620 China

**Keywords:** CRC, FOLFOX, High-salt diet, Tryptophan metabolism, Immune regulation

## Abstract

**Background:**

FOLFOX is the recommended chemotherapy regimen for colorectal cancer (CRC), but its response rate remains low. Our previous studies have established a close relationship between gut microbiota and the anti-CRC effect of FOLFOX, though the underlying mechanisms remain unclear. Diet has been confirmed as a key factor influencing gut microbiota, and high-salt diets, representative of western dietary habits, has been shown to affect gut microbiota, immune function, and the risk of developing CRC. However, the impact of high-salt diets on the anti-CRC efficacy of FOLFOX remains unstudied. Therefore, we aimed to investigate the effect and mechanism of high-salt diets on the anti-CRC effect of FOLFOX.

**Methods:**

We performed 16 S rRNA sequencing and T500 targeted metabolomics analysis on fecal samples from CRC patients and healthy adults. A CRC orthotopic xenograft mouse model was used to study the effect of a high-salt diet on FOLFOX’s anti-CRC efficacy. 16 S rRNA sequencing and non-targeted metabolomics were conducted on mouse fecal samples. Flow cytometry was used to assess immune cell infiltration in tumor and paracancerous tissues. A mouse macrophage conditioned medium system, with tryptophan metabolites, was employed to annotate the functional metabolites, followed by in vivo verification using the orthotopic xenograft mouse model.

**Results:**

The structure and metabolic profiles of gut microbiota are significantly different between 9 healthy adults and 6 CRC patients. A high-salt diet significantly reduced the efficacy of FOLFOX in mice, with notable changes in gut microbiota and related metabolites. Correlation analysis revealed a significant relationship between gut microbiota, tryptophan metabolites and FOLFOX efficacy. Flow cytometry indicated that a high-salt diet altered macrophage infiltration (CD45^+^F4/80^+^) in both the tumor and paracancerous tissues. In vitro experiments confirmed that the tryptophan metabolite SK reduced FOLFOX efficacy, while IPA enhanced it through macrophage-conditioned medium. In vivo, we verified that under a high-salt diet, SK inhibited the efficacy of FOLFOX, while IPA promoted it.

**Conclusion:**

A high-salt diet reduces the anti-CRC efficacy of FOLFOX through gut bacterial tryptophan metabolism mediated macrophage immunomodulation.

**Supplementary Information:**

The online version contains supplementary material available at 10.1186/s10020-025-01122-8.

## Introduction

Colorectal cancer (CRC) has an annual increase of over 500,000 new cases with more than 200,000 deaths (Sung et al. 2021), making it one of the most common lethal cancer worldwide (Siegel et al. 2022). Due to the insidious onset of CRC, a considerable number of newly diagnosed patients exhibit unresectable metastatic diseases, and over 40% of patients undergo disease recurrence or metastatic diseases after surgery (Gill et al. 2007). Therefore, chemotherapy has been the preferred therapeutic strategy against CRC. Fluorouracil, Calcium Folinate combined with different doses of Oxaliplatin, known as FOLFOX regimen, is the most recommended neoadjuvant chemotherapy for CRC (Gustavsson et al. 2015, (2020) 2020). However, the clinical response rate of FOLFOX is only 30–50% (Bokemeyer et al. 2009). It is urgent to reveal the underlying mechanism of limited FOLFOX efficacy and to investigate potential synergistic targets.

Gut microbiota is a microbial community inside gastrointestinal tract and is closely related to human health. Accumulating evidence confirmed that gut microbiota and derived metabolites are involved in the occurrence, development, and treatment of CRC (Fong et al. 2020; Chen et al. 2022; Bell et al. 2022; Peng et al. 2020). Our previous study showed that gut microbiota participates in the individualized efficacy of FOLFOX in CRC bearing mice, but the upstream mechanism remains largely unknown (Hou et al. 2021a, b). Various internal and external factors such as genetics, dietary habit, exercise and geographic location affect the distribution and function of gut microbiota. As one of the most significant factors in gut microbiota modulation, everyday diet received increasing attentions recently (Beam et al. 2021). And it is known that dietary habit plays important roles in chemotherapy response against cancer (Kuo et al. 2016).

The western diet represented by high salt and high fat is known to be related to various diseases (Manzel et al. 2014; Fiecke et al. 2023), such as cancer, autoimmune diseases, cardiovascular disease and chronic inflammation (He et al. 2020). High salt diet is also found to affect gut microbiota (Wilck et al. 2017), immunity (Rizvi et al. 2021), and closely related to the risk of developing CRC (Vernia et al. 2021). As reported, FOLFOX could interact with body immunity. It increases the number of cytotoxic CD8^+^ T lymphocytes in the tumor microenvironment and ultimately regulate anti-tumor immunity. And the use of immune checkpoint blockade (ICB) enhances the efficacy of FOLFOX (Guan et al. 2020). Considering the confirmed association between FOLFOX and microbiota, we wonder whether high salt diet is involved in the limited efficacy of FOLFOX and the underlying mechanism.

This study aims to examine the relationship between high salt diet and FOLFOX anti-cancer efficacy through microbiome and functional metabolomic analysis using a CRC orthotopic implantation mouse model. Meanwhile, spearman correlation analysis as well as MESA (metabolite sets enrichment analysis) pathway enrichment analysis were integrated with 16s rRNA sequencing and non-targeted/targeted metabolomic analysis to annotate key bacterial factors in high salt diet mediated FOLFOX efficacy attenuation. The involvement of immune regulation in this phenomenon was then investigated. Finally, an in vitro system of gut bacterial tryptophan metabolites conditioned macrophage culture was employed to elucidate the underlying mechanism. This study provides solid scientific evidence to explain the limited efficacy of FOLFOX against CRC, and proposes a novel synergistic strategy for FOLFOX anti-CRC therapy.

## Materials and methods

### Clinical samples

Clinical fecal samples (6 CRC patients and 9 Healthy adults, Suppl. Table S1), tumor and paired paracancerous tissues (10 CRC patients, Suppl. Table S2) were collected from Renmin Hospital of Huangpi District of Jianghan University (Wuhan, China). The experiment was approved by the Ethics Committee of School of Medicine of Jianghan University. Informed consent was obtained from all subjects and the experiments conform to the principles set out in the WMA Declaration of Helsinki and the Department of Health and Human Services Belmont Report.

### 16s rRNA gene sequencing analysis

Bacterial DNA extraction and quantification was performed as described previously (Hou et al. 2021a, b). The PCR products were detected by electrophoresis using agarose gel of 2% concentration. The highly variable regions (515 F and 806R) of the bacterial 16 S gene V4 were amplified by PCR. The products were sequenced on the NovaSeq6000 platform. Sequences analysis were performed by Uparse software (Uparse v7.0.1001, http://drive5.com/uparse/) (Edgar 2013). Sequences with ≥ 97% similarity were assigned to the same OTUs. Representative sequence for each OTU was screened for further annotation. OTUs abundance information were normalized using a standard of sequence number corresponding to the sample with the least sequences. α-diversity is applied in analyzing complexity of species diversity for a sample through 3 indices, including Chao1, Shannon, Simpson. All these indices in our samples were calculated with QIIME (V ersion 1.7.0). β-diversity analysis on unweighted unifrac (QIIME software) was used to evaluate differences of samples in species complexity. MetaStat analysis was applied to identify the differentially abundant taxa between the groups (it was considered statistically significant when *p* < 0.05).

### T500 targeted metabolomic analysis

CRC patients and Healthy adults’ fecal samples were detected by T500 targeted metabolomic analysis. Briefly, the samples were extracted by 70% methanol/water, seperated by Waters ACQUITY UPLC HSS T3 C18 (100 mm×2.1 mm i.d.ˈ1.8 μm) and detected using a QTRAP^®^ 6500 + LC-MS/MS System in both positive and negative ion mode. Solvent system: water with 0.05% formic acid (A), acetonitrile with 0.05% formic acid (B). Column temperature: 40 ℃; Injection volume: 2 µL; ESI+/- source temperature: 550 ℃; Interface voltage was 5.5 kV for positive mode and − 4.5 kV for negative mode. metabolites were annotated by comparing with the standard compounds with respect to retention time, accurate mass as well as mass spectra. Orthogonal partial least-squares-discriminant analysis (OPLS-DA) models and fold change were used to determine whether a feature is significantly different between the two groups or not. In order to avoid overfitting, a permutation test (200 permutations) was performed.

### Chemicals and reagents

5-FU was obtained from J&K (Beijing, China), Calcium Folinate was obtained from Tcichemicals (Shanghai, China) and Oxaliplatin were obtained from Accela ChemBio (Shanghai, China). FOLFOX injection was prepared according to our previous studies (Hou et al. 2021a, b). Tryptamine (TM), Indole (IN), Indole propionic Acid (IPA), Indole-3-lactic Acid (ILA), Indole-3-carboxaldehyde (I3A) and Skatole (SK) was purchased from Aladdin (Shanghai, China). Tryptophan (TP), Kynurenine (3HK) and 3-Indoleacetic Acid (IAA) was purchased from J&K (Beijing, China).

### Cell culture

Mice CRC cell line CT26 was obtained from the Cell Bank of the Institute of Biochemistry and Cell Biology, Chinese Academy of Sciences (Shanghai, China). Luci-CT26 was generated through transduction of lentiviral vectors encoding firefly luciferase (LV16-NC) purchased from GenePharma Co., Ltd. (Shanghai, China) supplied with 5 µg/mL polybrene (Sigma), and screened by and puromycin (5 µg/mL, Sigma-Aldrich). Mice monocytic cell line RAW264.7 were gift from Immunology Teaching and Research Section of Tongji Medical College of Huazhong University of Science and Technology (Hubei, China). All the cells were cultured in DMEM (Gibco, Grand Island, USA) with 10% Fetal Bovine Serum (FBS, Gibco), and incubated at 37 °C in a humidified atmosphere with 5% CO_2_.

### Animal experiments

Six to eight-week-old male BALB/c mice (20–22 g) were provided by the Hunan Slake Jingda Experimental Animal Co. Ltd. (Hunan, China) with the permission number SCXK (Xiang) 2019-0004. The study was conducted in accordance with the standards established by the Medical Ethics Committee of Jianghan University. All the mice were housed in temperature-controlled environment (24 ± 2 °C) under a 12/12 h dark/light cycle.

After acclimatization, mice were randomly divided into groups and fed with either Control diet (CD) or High salt diet (HSD; 8% NaCl) (day 1). Luci-CT26 tumor tissues were orthotopically inoculated at 1–2 cm below the cecum in mice to establish a CRC orthotopic implantation mouse model (day 21). Tumor formation was confirmed 5 days (day 26) after modeling via the in vivo spectral real-time imaging system (IVIS, USA), then the HSD mice were randomly divided into High salt group (HS, *n* = 10) and High salt combined FOLFOX group (HSF, *n* = 10); the CD mice were randomly divided into Model group (M, *n* = 10), FOLFOX group (MF, *n* = 10) and Control group (C, *n* = 10) without modeling. Fecal samples were collected at day 26 before FOLFOX treatment. On the day 27, FOLFOX (6 mg/kg Oxaliplatin followed by 50 mg/kg 5-FU and 90 mg/kg Calcium folinate in 2 h) was intraperitoneal (*i.p.*) administrated to HSF and FOLFOX group on a weekly basis (day 27&34)(Hou et al. 2021a, b). Tumor volume was monitored by in vivo spectral real-time imaging system (day 39) throughout the experiment. Relative Total Flux = Total Flux at day 39/ Total Flux at day 26. Inhibition Rate = 1-Relative Total Flux of the experimental group (MF/MSF)/ Relative Total Flux of the model group (M/MS), respectively. All the mice were sacrificed at day 40, the liver, spleen, paracancerous and tumor tissues were removed for further experiment.

For the in vivo validation experiment, the grouping, high-salt diet feeding, and modeling methods were consistent with the previous batch of animal experiments. After establishing the CRC orthotopic xenograft mouse model (Day 25), tumor formation was confirmed 5 days post-modeling (Day 29) using an in vivo spectral real-time imaging system (IVIS, USA). The HSD mice were then randomly divided into the following groups: high-salt group (HSD-M, *n* = 5), high-salt combined with FOLFOX group (HSD-MF, *n* = 5), high-salt with SK administration group (HSD-SKM, *n* = 5), high-salt with SK administration combined with FOLFOX group (HSD-SKMF, *n* = 5), high-salt with IPA administration group (HSD-IPAM, *n* = 5), and high-salt with IPA administration combined with FOLFOX group (HSD-IPAMF, *n* = 5). The CD (control diet) mice were randomly divided into the model group (CD-M, *n* = 5) and the FOLFOX group (CD-MF, *n* = 5). The dosage and frequency of FOLFOX administration followed the same protocol as the previous batch of animal experiments. The dosage of tryptophan metabolite SK was 20 mg/kg (Kim et al. 2020), administered once daily, while the dosage of tryptophan metabolite IPA was 25 mg/kg (Fang et al. 2022), also administered once daily. Tumor volume was monitored throughout the experiment using the in vivo spectral real-time imaging system (Day 43). The relative total flux wes calculated as: Relative total flux = total flux on Day 43 / total flux on Day 29. Inhibition rates were calculated using the following formula: Inhibition rate = 1 - (relative total flux of the experimental group (CD-MF/HSD-MF/HSD-SKMF/HSD-IPAMF) / relative total flux of the model group (CD-M/HSD-M/HSD-SKM/HSD-IPAM). All mice were euthanized on Day 43.

### Histopathology

Liver, spleen, paracancerous and tumor tissues were formalin fixed and paraffin embedded. Sections were then subjected for hematoxylin and eosin (HE) staining and immunohistochemistry (IHC) (Hou et al. 2018). Ki67 is a nuclear protein only expressed in proliferating cells, which has been confirmed correlating with the therapeutic outcomes of cancer patients, and applied as a critical tumor pathological grade and prognostic factor in many cancers (Luo et al. 2005). In the current study, tumor proliferation status was assessed by the percentage of positively Ki67 stained cells. Scoring was defined as the proportion of Ki67 positively stained cells in total tumor cells (Select 5 fields of view randomly) (Minckwitz et al. 2013).

### Non-targeted metabolomic analysis

Fecal samples detected by non-target metabolomic analysis were based our previous studies (LC-MS/MS) (Hou et al. 2023). Briefly, the samples were extracted by 70% methanol/water and analyzed using a LC-MS system (Triple TOF-6600 mass spectrometer). Compound separation was performed on a Waters ACQUITY UPLC HSS T3 C18 (1.8 μm, 2.1 mm*100 mm); column temperature: 40 ^∘^C; flow rate: 0.4 mL/min; injection volume: 2 µL; solvent system: (A) water (0.1% formic acid); (B) acetonitrile (0.1% formic acid); The column was eluted with 5% mobile phase B at 0 min followed by a linear gradient to 90% mobile phase B over 11 min, held for 1 min, and then come back to 5% mobile phase B within 0.1 min, held for 1.9 min. The original data file acquisited by LC-MS was converted into mzML format by ProteoWizard software. Metabolites were annotated by comparing the m/z values, formulae and the MS/MS fragmentations with to online databases. The data was unit variance scaled before unsupervised PCA. For two-group analysis, differential metabolites were determined by VIP (VIP > 1), Fold Change > 2 or < 0.5 and *p* value (*p* value < 0.05, Student’s t test). MSEA was applied to identify the differentially levels of metabolic pathways between C and M mice.

### Macrophage conditioned medium collection and application

Firstly, 1.2 × 10^6^ RAW264.7 macrophages were seeded into a culture dish. After adhering to the wall, the cells were co-cultured with tryptophan and derived metabolites (including TP, TM, IN, SK, ILA, IPA, I3A, 3HK and IAA) for 24 h. The medium was centrifuged at 3500 g for 10 min, the supernatant was collected and stored at − 80 °C for further experiments. In application, using 1:1 mixture of conditioned medium and conventional medium to CT26 cells (Zhou et al. 2019; Chen et al. 2023).

### Western blot, RT-qPCR and cell proliferation assays

The western blot, RT-qPCR and cell proliferation assays were conducted based on our previous studies (Hou et al. 2018). Briefly, the PCNA levels in the tumor tissues were evaluated by western blot using PCNA antibodies. The relative mRNA levels of IL-1β and TNF-α were evaluated by RT-qPCR. The cell proliferation assays were performed by MTT and Colony formation assay. FOLFOX concentrations (µM) of Oxaliplatin: Fluorouracil: Calcium folinate = 0.5: 0.5: 0.9 (F1), 1: 0.5: 0.9 (F2), 0.5: 1: 1.8 (F3) were finally selected for MTT and 0.1: 0.1: 0.18 (F1), 0.2: 0.1: 0.18 (F2), 0.1: 0.2: 0.36 (F3) were finally selected for Colony formation assay. Q value as an evaluation index for synergistic or anergic effect on drug combination, Q < 0.85 antagonistic, 0.85 ≤ Q < 1.15 additive, Q ≧ 1.15 synergistic (Eljack et al. 2022).

### Statistical analysis

Spearman’s correlation analysis was applied to test the correlation between fecal bacteria/metabolite intensities and efficacy evaluation indicators (GraphPad Prism 10 software, GraphPad Software Inc., La Jolla, CA, USA). Data analysis and graphing were performed by GraphPad Prism 10 software. The results were presented as mean ± SD, independent unpaired two-tailed Student’s *t* test was performed to evaluate the differences between two groups, unless otherwise specified.

## Results

### The structure and metabolic profile of gut microbiota is changed in CRC patients

**Fig. 1:**
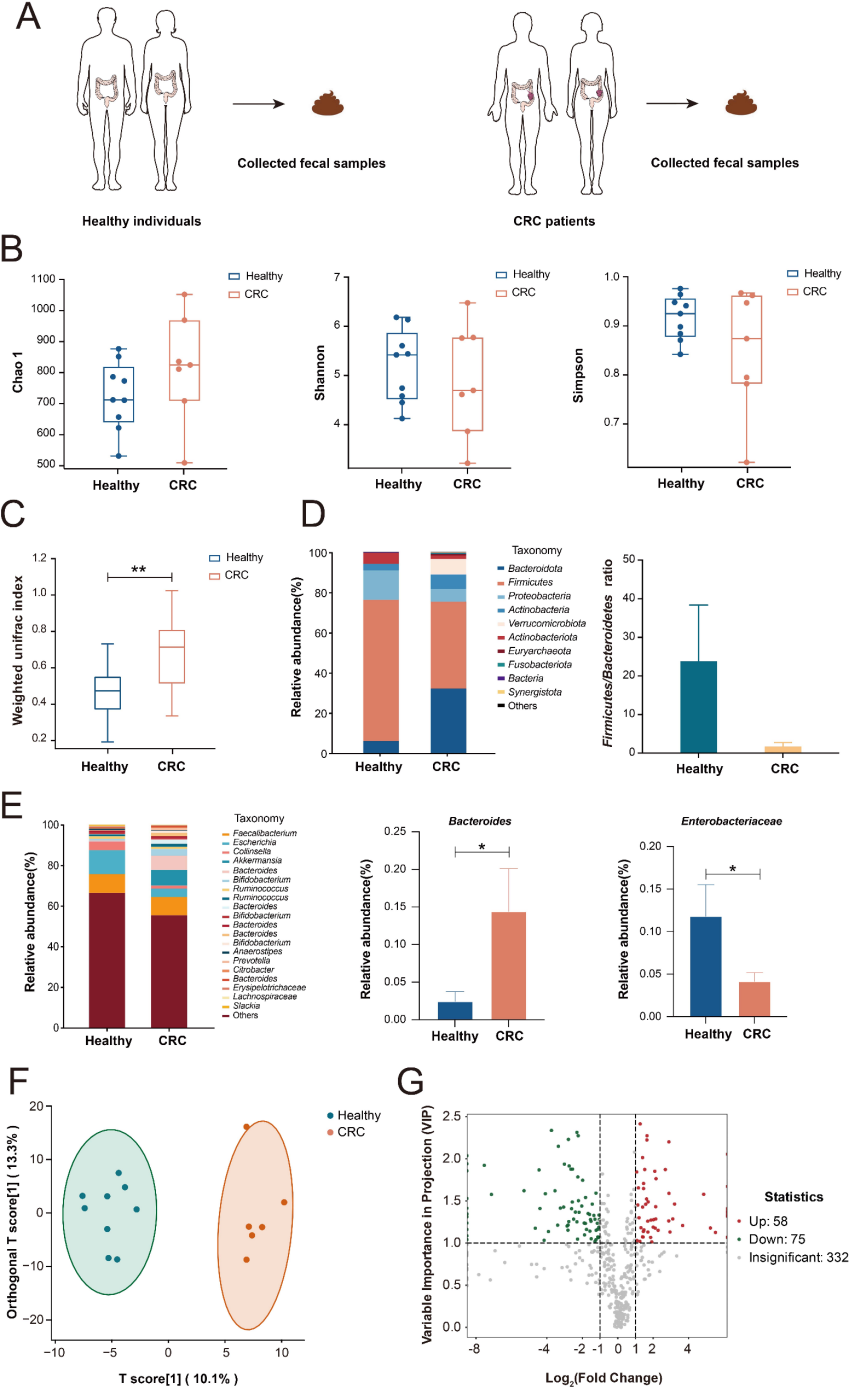
16s rRNA sequencing and T500 targeted metabolomics analysis of fecal samples from CRC patients and Healthy volunteers. (**A**) Fecal samples collected from 6 CRC patients and 9 Healthy adults. Analysis results of α (**B**) and β (**C**) diversity in fecal samples from CRC patients and Healthy adults. (**D**) Stacked charts were compiled for the top 10 bacterial phyla in CRC patients and Healthy adults, and the enterotype of the two groups were calculated separately. (**E**) Stacked graph for statistics of the top 20 bacterial genera in CRC patients and Healthy adults, and the abundance of *Bacteroides* and *Enterobacteriaceae* of the two groups. (**F**) The fecal metabolism profile of CRC patients and Healthy adults demonstrated by OPLS-DA (R^2^X = 0.234, R^2^Y = 0.956, Q^2^ = 0.452). (**G**) Volcano plots of differential metabolites with VIP > 1 and Fold Change > 2 or < 0.5 criteria between the two groups. *p < 0.05, **p < 0.01

To investigate the involvement of gut microbiota in CRC, we collected fecal samples from Healthy adults (*n* = 9) and CRC patients (*n* = 6) for 16s rRNA sequencing and T500 targeted metabolomic analysis (Fig. 1A). The differences in α and β diversity were analyzed to assess variations in the composition and structure of the gut microbiota. Although the α-diversity indexes (Chao1, Shannon and Simpson) were not significantly changed (Fig. 1B), the overall gut bacterial structure and composition was significantly different between Healthy individuals and CRC patients as indicated by β-diversity index (Fig. 1C). Specifically, the enterotype (*Firmicutes*/*Bacteroidetes*) was prominently changed in CRC group when compared with Healthy group (Fig. 1D). At genus level, Metastats analysis revealed that the abundance of *Bacteroides et al.* increased significantly while *Enterobacteriaceae et al.* decreased significantly in feces from CRC patients (Fig. 1E and Suppl. Fig. S1). These findings indicate that the composition and structure of gut microbiota in CRC patients differ notably from those in healthy individuals, suggesting that the alterations in gut microbiota composition may contribute to the progression of CRC.

The abundance and composition of bacterial metabolites is important in gut microbiota function (Nicolas and Chang 2019). We compared the fecal metabolic profiles of Healthy adults and CRC patients using T500 targeted metabolomic analysis. As shown in Fig. 1F, OPLS-DA analysis suggested significant differences in the total metabolic profiles of fecal samples between the two groups. After data processing, 80 differential metabolites (VIP value > 1 and Fold Change > 2 or < 0.5) were annotated (Fig. 1G) mainly including tryptophan and related metabolites, short chain fatty acids (SCFAs), secondary bile acids, amino acids, and short peptides (Suppl. Table S3). Taken together, the modified distribution and metabolic profile of gut microbiota suggests that gut microbiota and related metabolites are involved in the development of CRC.

### High salt diet inhibits the efficacy of FOLFOX in vivo

**Fig. 2:**
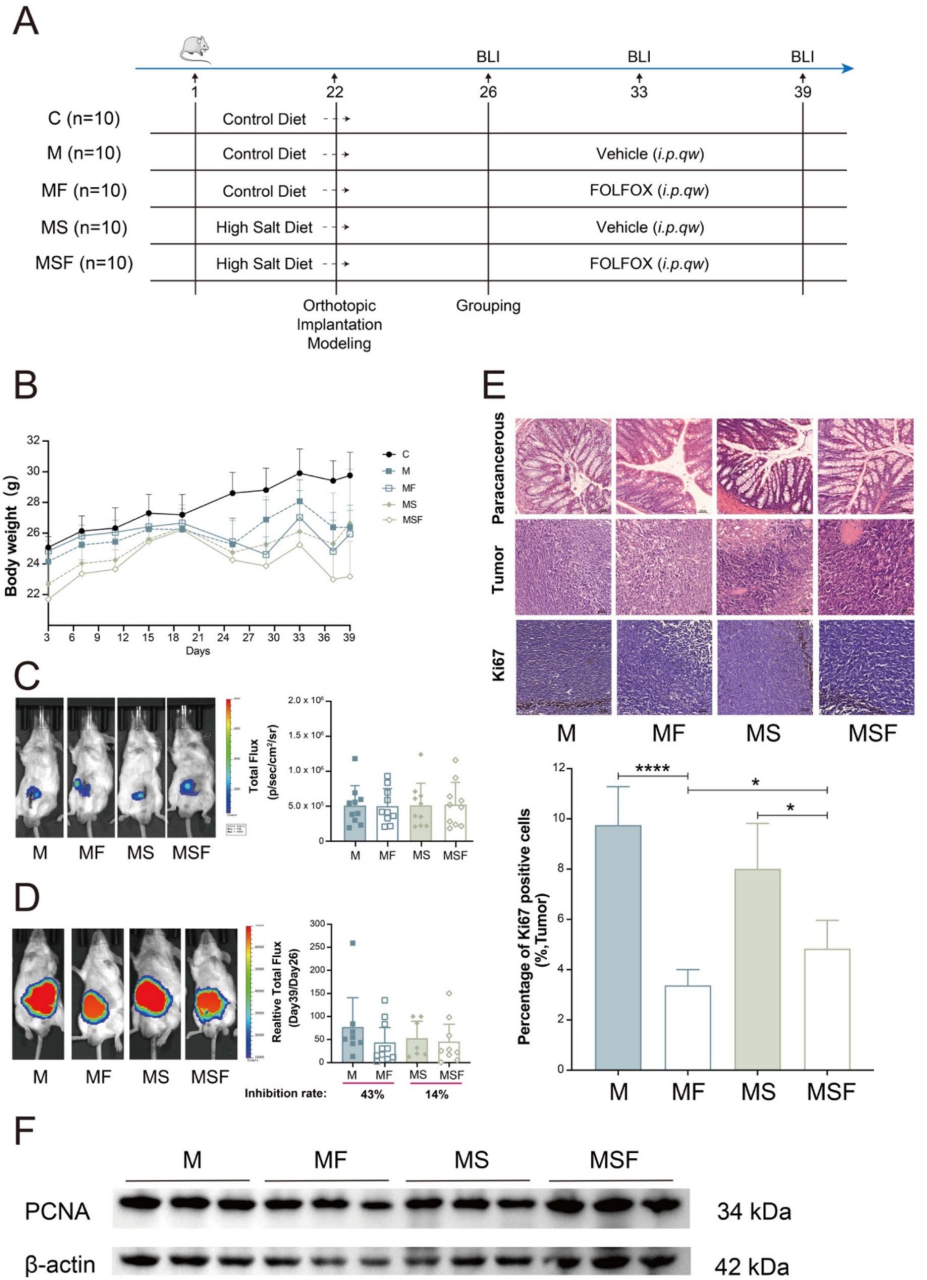
High salt diet inhibits the efficacy of FOLFOX in vivo. (**A**) Schematic diagram of animal experiments. The High salt diet group (MS + MSF) was intervened with high salt diet for the whole experiment. (**B**) Change of mice body weight during the experiment. (**C**) Representative bioluminescence images and total flux at day 26 (grouping). (**D**) Representative bioluminescence images, relative total flux and tumor inhibition rate at day 39 (the end of the experiment) (**E**) HE staining and immunohistochemistry (Ki67) of mice in each group. (**F**) Expression of PCNA in tumor tissue of each group. C, Control. M, Model. MF, FOLFOX group. MS, High salt diet group. MSF, High salt diet combined with FOLFOX group. * *p* < 0.05, **** *p* < 0.0001

Dietary intervention is an important strategy for gut microbiota regulation including the composition, structure, derived metabolites and related inflammatory responses (García-Montero et al. 2021). High salt diet, as an important component of western diets, has been found to affect the effectiveness of various anti-tumor treatments (Deng et al. 2019; Khandekar et al. 2022). In order to understand the potential involvement of high salt diet in FOLFOX anti-cancer efficacy, we constructed a CRC orthotopic implantation mouse model based on luciferase labeling CT26 cells (Fig. 2A). Control diet (M group and MF group) or high salt diet (MS group and MSF group) were given during the whole experiment (day 1 - day 39), and fecal samples were collected the day before FOLFOX treatment (day 26) for 16s rRNA sequencing and non-targeted metabolomic analysis. Consistent with previous studies (He et al. 2020; Kumar et al. 2022; Pajtók et al. 2021), diet consumption significantly increased in MS and MSF groups while high salt diet did not affect mice body weight (Fig. 2B and Suppl. Fig. S2). And FOLFOX treatment result in decreased body weight in MF and MSF groups. Before the first FOLFOX administration (at day 26), the total flux of CRC was measured in all the tumor bearing mice. According to these data, experimental mice were randomly divided into different groups for the following FOLFOX or vehicle treatments (Fig. 2C). The tumor inhibition rate at day 39 determined by relative total flux revealed that high salt diet reduced the anti-cancer ability of FOLFOX from 43 to 14% (Fig. 2D). Further investigation with cancer tissues showed that the down regulation of Ki67 as well as PCNA after FOLFOX treatment were significantly reversed in high salt diet groups (Fig. 2E-F). In addition, histopathological analysis indicates that mice have no severe toxic effect in liver and spleen under current experimental conditions (Suppl. Fig. S3). These data suggest that high salt diet inhibits the anti-CRC efficacy of FOLFOX in vivo*.*

### High salt diet significantly changed the structure and metabolic profile of gut microbiota on CRC bearing mice

**Fig. 3:**
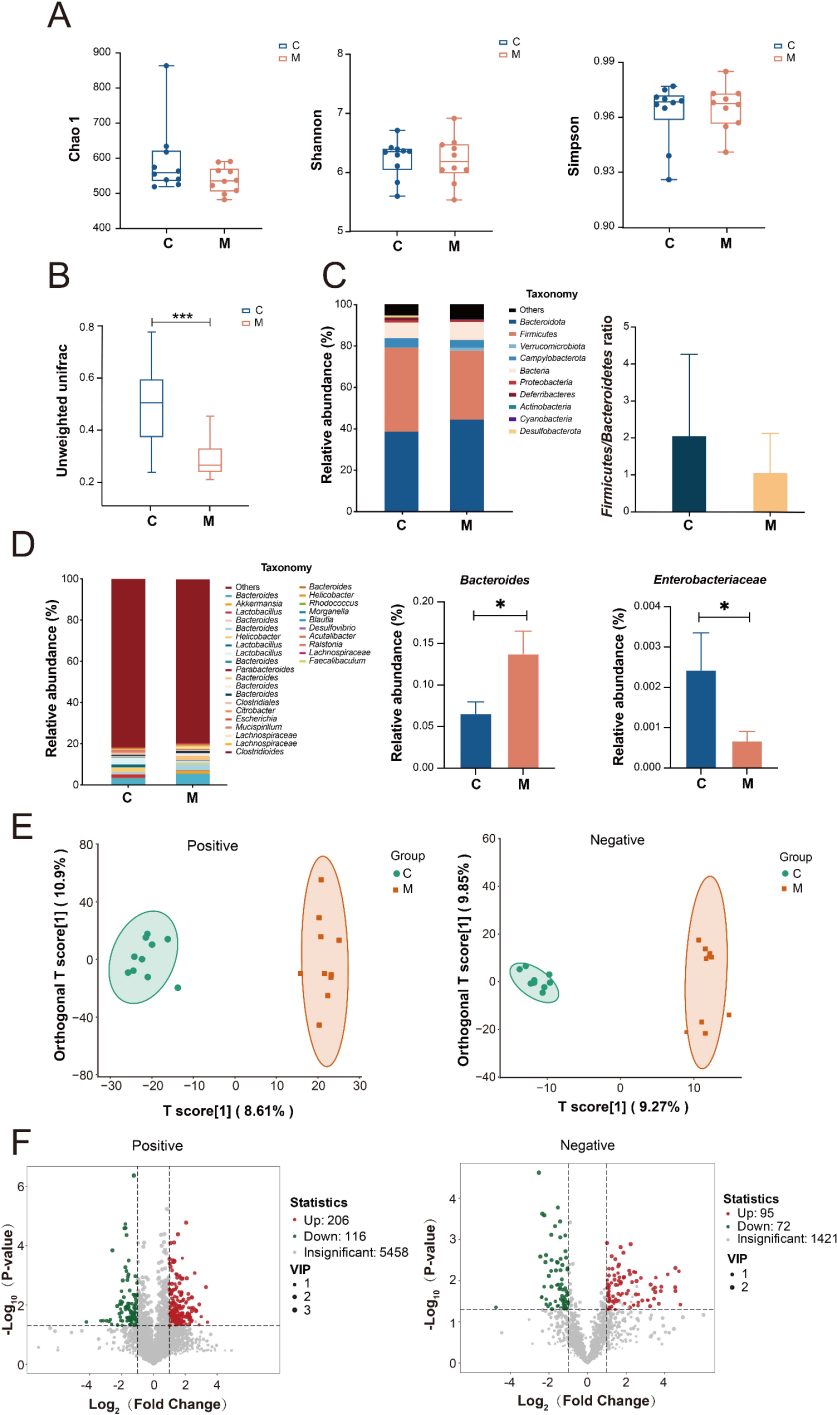
16s rRNA sequencing and non-targeted metabolomics analysis of fecal samples from Control and Model group. The α-diversity indexes of Chao1, Shannon and Simpson (**A**) and the β (**B**) diversity in fecal samples from C and M mice. (**C**) Stacked charts were compiled for the top 10 bacterial phyla with relative abundance in C and M mice, and the ratios of two groups of *Bacteroidetes* to *Firmicutes* (i.e. enterotype) were calculated separately. (**D**) Stacked graph for statistics of the top 30 bacterial genera with relative abundance in C and M mice, and the abundance of *Bacteroides* and *Enterobacteriaceae* of the two group. (**E**) The fecal metabolism profile of C and M mice demonstrated by OPLS-DA LC–MS (+) (R^2^X = 0.195, R^2^Y = 0.981, Q^2^ = 0.545) and LC–MS (-) (R^2^X = 0.191, R^2^Y = 0.987, Q^2^ = 0.539). (**F**) Volcano plots of differential metabolites with VIP value > 1 and *p* value < 0.05 and Fold Change > 2 or < 0.5 criteria. C, Control. M, Model. Positive, positive ion mode. Negative, negative ion mode. *** *p* < 0.001

To investigate whether gut bacteria contributes to the attenuation of FOLFOX efficacy by high salt diet, 16s rRNA sequencing and non-target metabolomic analysis on pre-FOLFOX fecal samples of different groups were conducted. Consistent with our findings from patients’ fecal samples, CRC development in mice significantly changed the community structure of gut microbiota (β-diversity) while the diversity and richness (α-diversity) were not altered (Fig. 3A-B). We also observed decreased ratio of *Firmicutes*/*Bacteroidetes* (enterotype), significantly increased relative abundance of *Bacteroides et al.* and significantly decreased *Enterobacteriaceae et al.* between C and M mice (Fig. 3C-D and Suppl. Fig. S4), indicating the bacterial distribution was prominently changed. Meanwhile, non-targeted metabolomic analysis revealed significant changes of gut microbiota metabolic profile in mice with CRC under both positive and negative iron detection mode (Fig. 3E). And after metabolite annotation, 127 differential metabolites with VIP value > 1 and Fold Change > 2 or < 0.5 and *p* value < 0.05 were identified (Fig. 3F and Suppl. Table S4). These differential metabolites mainly include amino acids and derivatives, as well as hormones and related substances. Further MESA pathway enrichment analysis suggested that levels of bacterial tryptophan metabolism were significantly different between C and M mice (Suppl. Fig. S5). These results further indicates that gut microbiota and related metabolites are involved in the occurrence and development of CRC, and the current orthotopic implantation tumor mice model properly simulate the clinical situation.

**Fig. 4:**
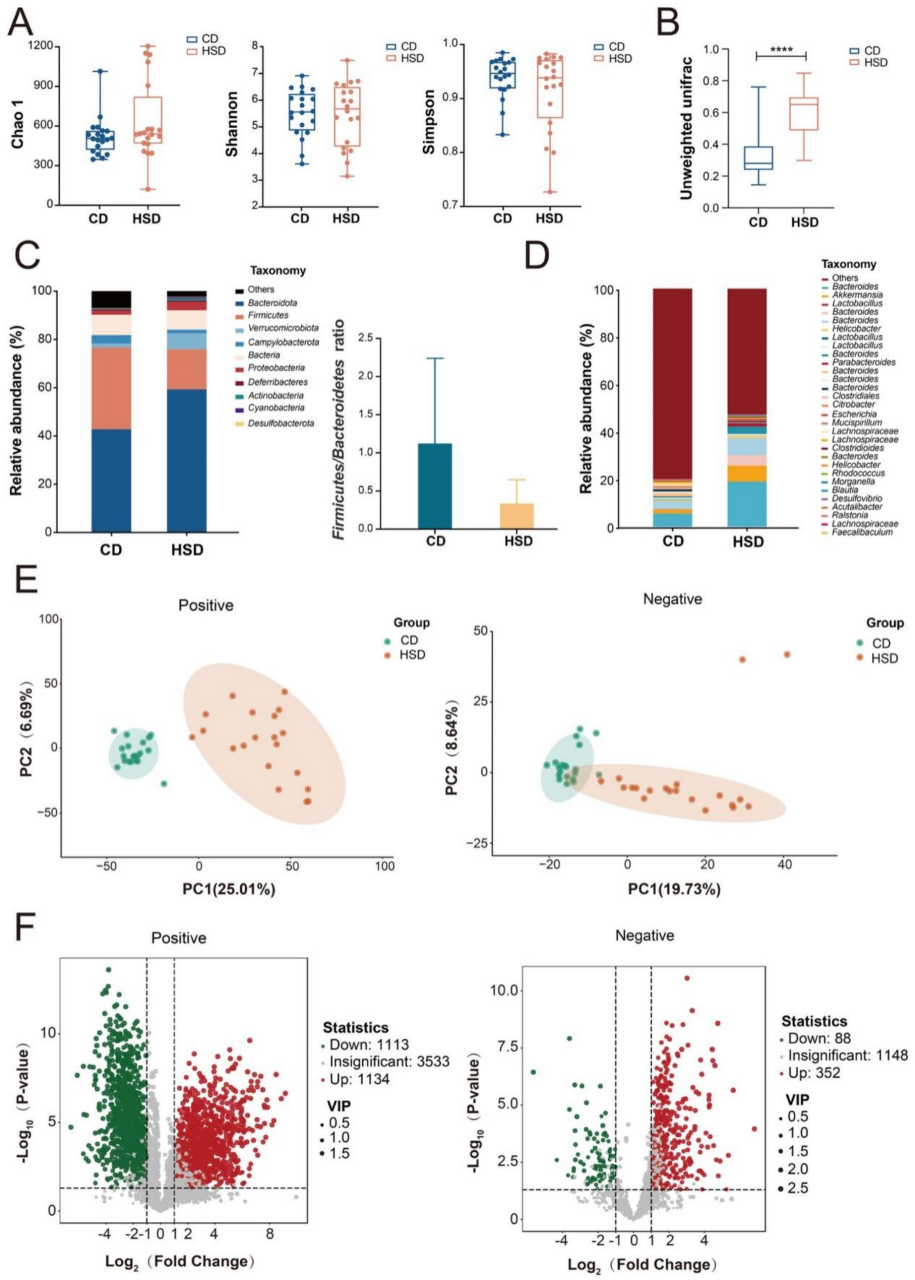
16s rRNA sequencing and non-targeted metabolomics analysis of fecal samples from CD mice and HSD mice. The α-diversity indexes of Chao1, Shannon and Simpson (**A**) and the β (**B**) diversity in fecal samples from CD mice and HSD mice. (**C**) Stacked charts were compiled for the top 10 bacterial phyla with relative abundance in CD mice and HSD mice, and the ratios of two groups of *Bacteroidetes* to *Firmicutes* (i.e. enterotype) were calculated separately. (**D**) Stacked graph for statistics of the top 30 bacterial genera with relative abundance in CD mice and HSD mice. (**E**) The fecal metabolism profile of CD mice and HSD mice demonstrated by PCA (Principal Component Analysis). (**F**) Volcano plots of differential metabolites with VIP value > 1 and *p* value < 0.05 and Fold Change > 2 or < 0.5 criteria. Positive, positive ion mode. Negative, negative ion mode. CD (M + MF, *n* = 20). HSD (MS + MSF, *n* = 20)

We subsequently focused on high salt diet’s effect by comparing control diet mice (CD including M and MF groups) to high salt diet mice (HSD including MS and MSF groups). Interestingly, high salt diet also did not change the α-diversity of tumor bearing mice (Fig. 4A) while the β-diversity was significantly changed according to 16s rRNA sequencing analysis (Fig. 4B). Meanwhile, data showed that high salt diet significantly reduced the ratio of *Firmicutes*/*Bacteroidetes*, and the relative abundance of *Bacteroidetes* was significantly increased from HSD mice. This specific distribution of gut microbiota is consistent with the decreased ratio of *Firmicutes*/*Bacteroidetes* from CRC patients & mice (Figs. 1D and 3C), suggesting a negative role of high salt diet in CRC (Fig. 4C-D).

Further PCA analysis of non-targeted metabolomics revealed a significantly altered metabolic profile of gut microbiota between CD and HSD mice (Fig. 4E). And 130 differential metabolites with VIP value > 1 and Fold Change > 2 or < 0.5 and *p* value < 0.05 were annotated (Fig. 4F and Suppl. Table S5), which mainly include oligopeptide, amino acids and derivatives, fatty acyls, etc. In conclusion, these analyses suggest that gut microbiota and related metabolites may be involved in the attenuated anti-cancer effect of FOLFOX induced by high salt diet.

### High salt diet intervention decrease FOLFOX efficacy through immune regulation mediated by gut microbiota tryptophan metabolism

**Fig. 5:**
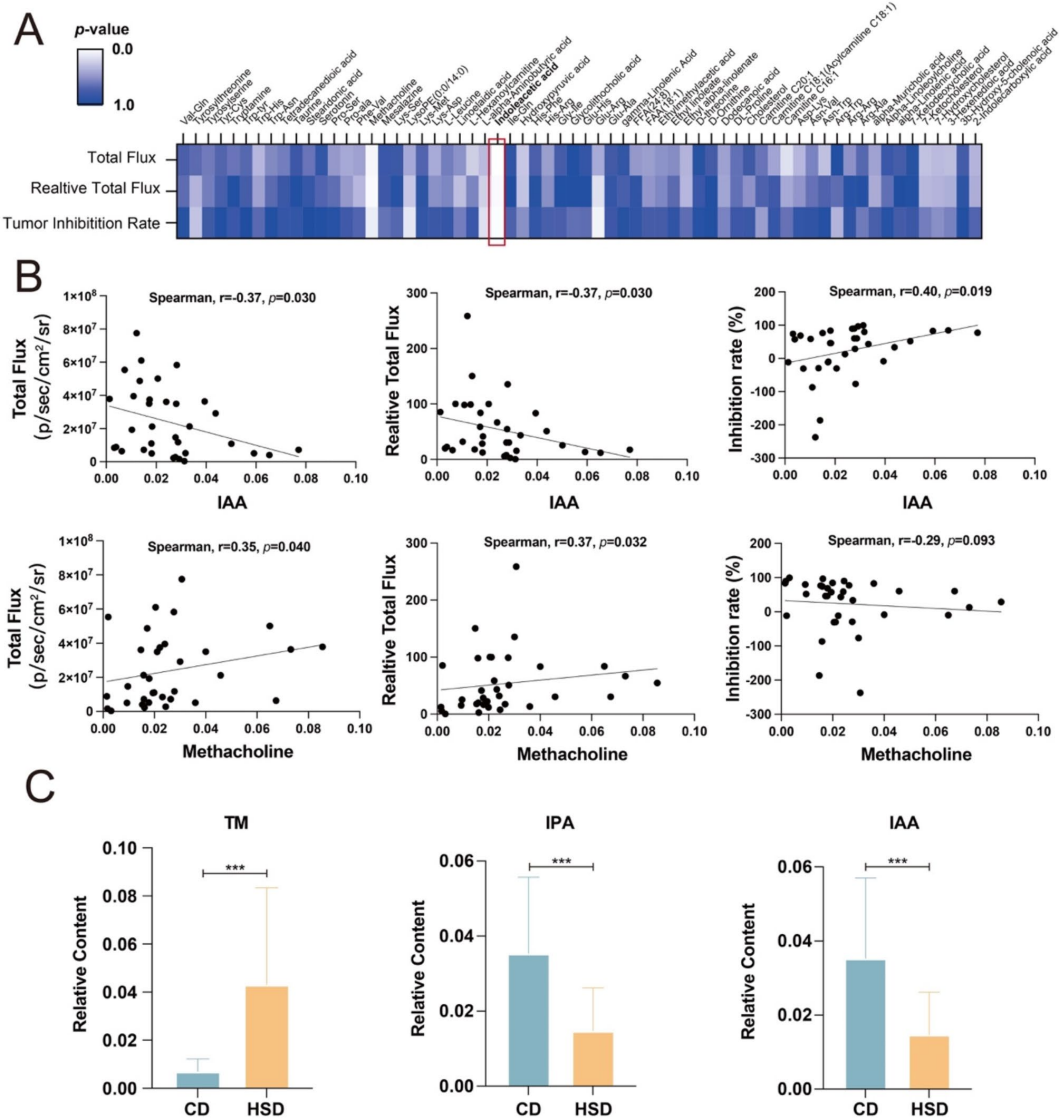
The abundance of bacterial tryptophan metabolism is significantly correlated with the efficacy of FOLFOX in CRC. (**A**-**B**) Spearman correlation analysis was conducted between the differential metabolites of all CRC bearing mice (M + MF + MS + MSF, *n* = 40) and the pharmacodynamic evaluation index (Total flux, relative total flux and tumor inhibition rate). (**C**) The significantly changed tryptophan metabolites between CD and HSD mice. TM, Tryptamine; IPA, 3-Indole propionic acid; IAA, 3-Indoleacetic acid. *** *p* < 0.001

To identify the responsible gut microbiota and/or derived metabolites in the decreased FOLFOX efficacy led by high salt diet, we conducted spearman’s correlation analysis between differential bacteria/metabolites and chemotherapeutic efficacy evaluation index (total flux, relative total flux and tumor inhibition rate) among the CD and the HSD groups (*n* = 40). As shown in Suppl. Fig. S6 and Fig. 5A-B, we did not observe any correlation between gut bacteria and chemotherapeutic efficacy evaluation index, but we found the abundance of IAA and Methacholine was significantly correlated with pharmacodynamic evaluation indexes of FOLFOX, and IAA shows higher significance and correlation index (r). We investigated the abundance of tryptophan metabolites detected in the feces of two groups and found that the level of bacterial tryptophan metabolites TM, IPA and IAA were significantly changed between CD and HSD mice (Fig. 5C and Suppl. Table S5). Considering the different metabolic activity of tryptophan metabolism between the gut microbiota of C and M mice (Suppl. Fig. S5), we speculate that bacterial tryptophan metabolism is important in high salt diet related reduced FOLFOX efficacy.

Subsequently, we investigated whether bacterial tryptophan metabolism could affect FOLFOX efficacy directly in vitro. Different FOLFOX concentration that were used in the following experiment were determined by MTT assay and referring to as F1, F2 and F3 (Suppl. Fig. S7A). Tryptophan (TP) and its bacterial metabolites (TM, IN, SK, ILA, IPA, I3A, 3HK and IAA) (Suppl. Fig. S8) (Agus et al. 2018) were directly combined with FOLFOX in CT26 cells to investigate the effect of tryptophan metabolites on the therapeutic effect of FOLFOX. Q value was selected as an evaluation index for synergistic or antergic effect on CT26 cell proliferation after treated by FOLFOX combined with tryptophan metabolites (Eljack et al. 2022). As a result, we observed that tryptophan metabolites do not significantly affect the efficacy of FOLFOX (0.85 ≤ Q < 1.15) (Suppl. Fig. S9 and Suppl. Table S6). Therefore, we consider whether tryptophan metabolites exert their effects through other mechanisms.

**Fig. 6:**
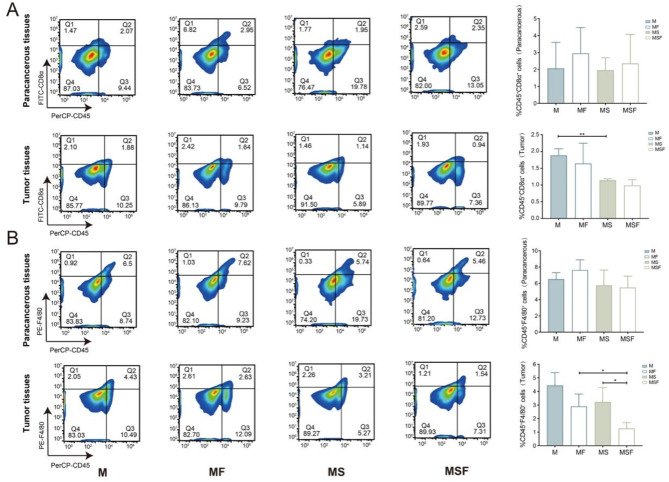
High salt diet decreased immune infiltration status of tumor and paracancerous tissues in CRC mice. Flow cytometry was applied to detect the infiltration status of cytotoxic T cells (**A**) and macrophages (**B**) of M, MF, MS and MSF mice in tumors and paracancerous tissues. * *p* < 0.05, ** *p* < 0.01

As reported, tryptophan bacterial metabolism could affect tumor immune microenvironment by lymphocytes recruitment and inflammatory factors secretion (He et al. 2022; Wu et al. 2021). Using flow cytometry, we examined the immune infiltration status of cytotoxic T cells (CD45^+^CD8^+^) and macrophages (CD45^+^F4/80^+^) in tumors and paracancerous tissues. As shown in Fig. 6, high salt diet (MSF) can reduce the levels of cytotoxic T cells and macrophage infiltration in tumor and paracancerous tissues when compared to control diet mice (MF) after FOLFOX treatment. And the infiltration level of macrophages in tumor tissue of MSF group mice was significantly lower than that of MF group. This indicates that a high salt diet can alter the tumor immune microenvironment, and macrophages may be the key to reducing the efficacy of FOLFOX in high salt diet.

### Tryptophan bacterial metabolites IN, I3A, IPA, IAA affect the anti-CRC effects of FOLFOX through macrophages

**Fig. 7:**
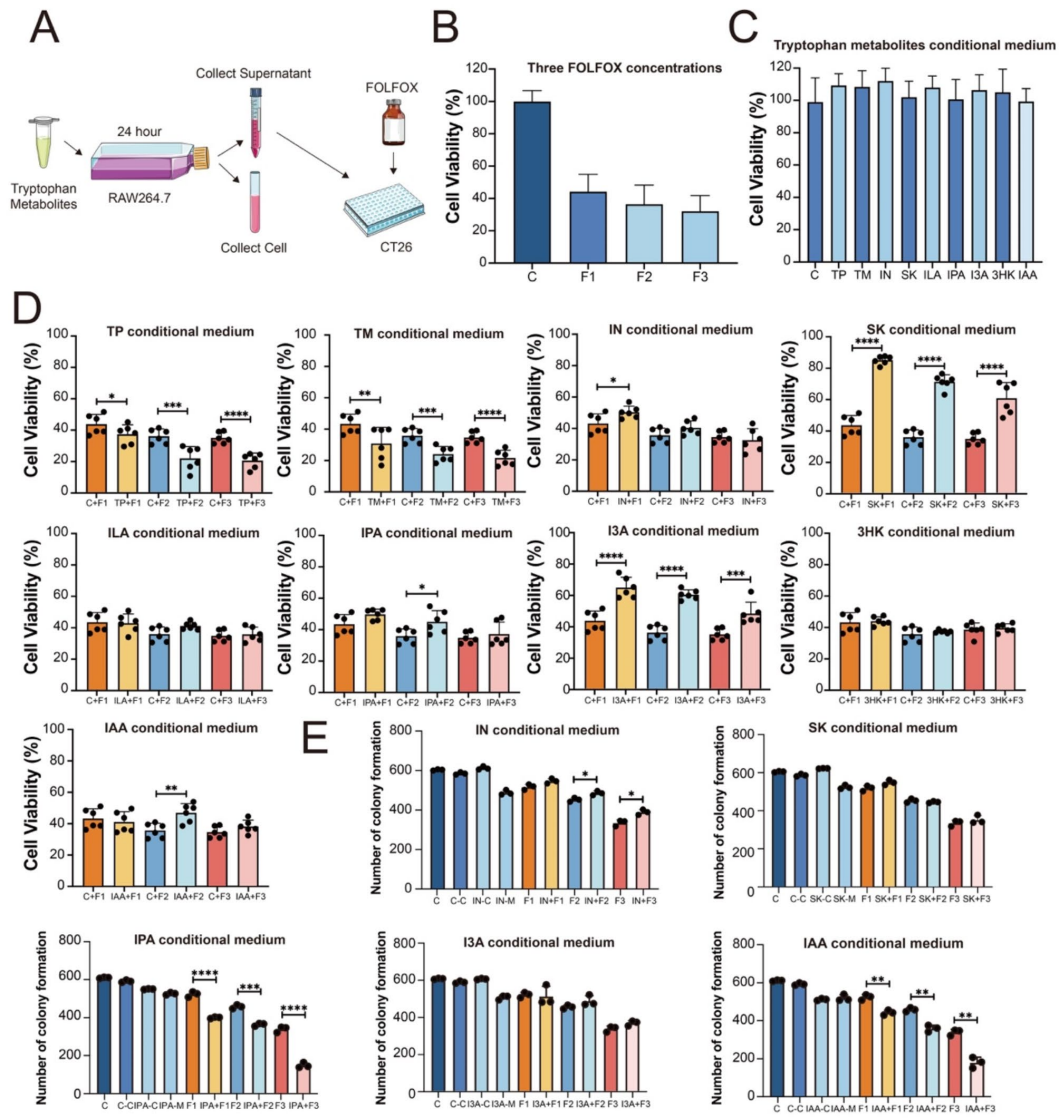
Effect of macrophage conditioned medium treated with different tryptophan metabolites on the anti-proliferative effect of FOLFOX. (**A**) Schematic diagram illustrating the preparation of macrophage conditioned medium with tryptophan metabolites. (**B**) Concentrations of FOLFOX used for combined treatment. (**C**) Evaluation of the impact of tryptophan metabolite conditioned medium alone on the anti-CRC efficacy of FOLFOX in vitro. (**D**) MTT assays results showing the effect of tryptophan metabolite conditioned medium on the anti-CRC efficacy of FOLFOX in vitro. (**E**) Colony formation assay results showing the effect of tryptophan metabolite conditioned medium on the anti-CRC efficacy of FOLFOX in vitro. C, Control medium. C-C, Control conditioned medium. IN-C, IN treatment conditioned medium. IN-M, IN medium. SK-C, SK treatment conditioned medium. SK-M, SK medium. IPA-C, IPA treatment conditioned medium. IPA-M, IPA medium. I3A-C, I3A treatment conditioned medium. I3A-M, I3A medium. IAA-C, IAA treatment conditioned medium. IAA-M, IAA medium.* *p* < 0.05, ** *p* < 0.01, *** *p* < 0.001, **** *p* < 0.0001

A further examination of clinical samples revealed that macrophage levels were significantly decreased in CRC when compared to paracancerous tissues (Suppl. Fig. S10). Since high salt diet al.so decrease macrophage infiltration in mice after FOLFOX treatment, we subsequently focused on the effect of macrophage regulation. We established a tryptophan metabolite conditioned cell culture medium system for mouse macrophages (RAW264.7) (Fig. 7A). TP and its bacterial metabolites were separately supplemented into the culture medium of RAW264.7 (Agus et al. 2018). We first investigated the effect of tryptophan metabolites on the production of inflammatory factors in macrophages. We examined the intracellular levels of IL-1β and TNF-α of RAW264.7 exposed to tryptophan and its metabolites by RT-qPCR. As shown in Fig. S11, tryptophan metabolites TP, TM, and ILA reduce the generation of IL-1β and TNF-α in macrophages; IN, SK, IPA, I3A, 3HK, and IAA increase the generation of IL-1β and TNF-α in macrophages, among which IN, SK, IPA, I3A were significantly, indicating that bacterial tryptophan metabolism is involved in immune regulation.

The above conditioned medium of RAW264.7 were collected and supplement into CT26 combined with FOLFOX to further test its effect on the anti-cancer efficacy of FOLFOX (Fig. 7A), and selected Q value as an evaluation index for synergistic or antergic effect on CT26 cell proliferation after treated by FOLFOX combined with conditioned medium. Above all, tryptophan metabolite conditioned medium alone did not affects the proliferation of CRC cells (Fig. 7B-C). Whereas the conditioned medium of IN, SK, IPA, I3A and IAA significantly attenuated FOLFOX efficacy (Q < 0.85/*p* < 0.05) (Fig. 7D; Table 1). And the conditioned medium of TP and TM increased the anti-cancer efficacy of FOLFOX in vitro (Q ≥ 1.15). Considering the increased secretions of IL-1β and TNF-α induced by IN, SK, IPA, and I3A in macrophages (Suppl. Fig. S11) and the significant association between IAA abundance with pharmacodynamic evaluation indexes of FOLFOX (Fig. 5B), we then used the conditioned medium of these 5 metabolites (IN, SK, IPA, I3A, IAA) to verify results in colony formation assay. Results confirmed that long term treatment (8 days) of the conditioned medium of IN, SK, I3A combined with different concentrations of FOLFOX (Suppl. Fig. S7B) inhibited the anti-cancer effect of FOLFOX (Q < 0.85) (Table 2). Interestingly, while exhibited antergic effect in short term exploration (MTT, Fig. 7D), IPA and IAA significantly promoted the effect of FOLFOX (Fig. 7E and Suppl. Fig. S12 and Table 2), suggested a transformed effect of IPA and IAA between short term and long-term exposure which deserves further exploration. Therefore, our findings demonstrate that a high-salt diet suppresses the anti-CRC efficacy of FOLFOX through immune regulation induced by gut bacterial tryptophan metabolism. Based on these results, SK and IPA were selected for further in vivo investigation.

**Table 1:** Q value of tryptophan metabolite conditioned medium combined with three concentrations of FOLFOX by MTT

No.	Tryptophan metabolite		F1	F2	F3
1		TP	1.22	1.31	1.23
2		TM	1.33	1.26	1.2
3		IN	0.89	0.92	0.97
4		SK	0.31	0.47	0.58
5		ILA	1.03	0.94	0.95
6		IPA	0.87	0.81	0.92
7		I3A	0.64	0.65	0.67
8		3HK	1.05	1.00	0.93
9		IAA	0.96	0.84	0.89

Q < 0.85 is antagonistic, 0.85 ≤ Q < 1.15 is additive, and Q ≥ 1.15 is synergistic. Nine tryptophan metabolites: TP: Tryptophan, 50 µM; TM: Tryptamine, 5 µM; IN: Indole, 500 µM; SK: Skatole, 100 µM; ILA: Indole-3-Lactic Acid, 800 µM; IPA: 3-Indole propionic acid, 500 µM; I3A: Indole-3-carboxaldehyde, 50 µM; 3HK: Kynurenine, 500 µM; IAA: 3-Indoleacetic acid, 1000 µM

**Table 2:** Q value of tryptophan metabolite conditioned medium combined with three FOLFOX drug concentrations by colony formation assay

No.	Tryptophan metabolite		F1	F2	F3
1		IN	0.61	0.76	0.78
2		SK	0.64	1.09	0.93
3		IPA	1.46	1.23	1.47
4		I3A	0.85	0.72	0.84
5		IAA	0.96	1.09	1.24

Q < 0.85 is antagonistic, 0.85 ≤ Q < 1.15 is additive, and Q ≥ 1.15 is synergistic. Nine tryptophan metabolites: IN: Indole, 500 µM; SK: Skatole, 20 µM; IPA: 3-Indole propionic acid, 100 µM; I3A: Indole-3-carboxaldehyde, 20 µM; IAA: 3-Indoleacetic acid, 200 µM

### The tryptophan metabolite SK and IPA are key molecules in the reduction of FOLFOX efficacy by a high-salt diet

**Fig. 8:**
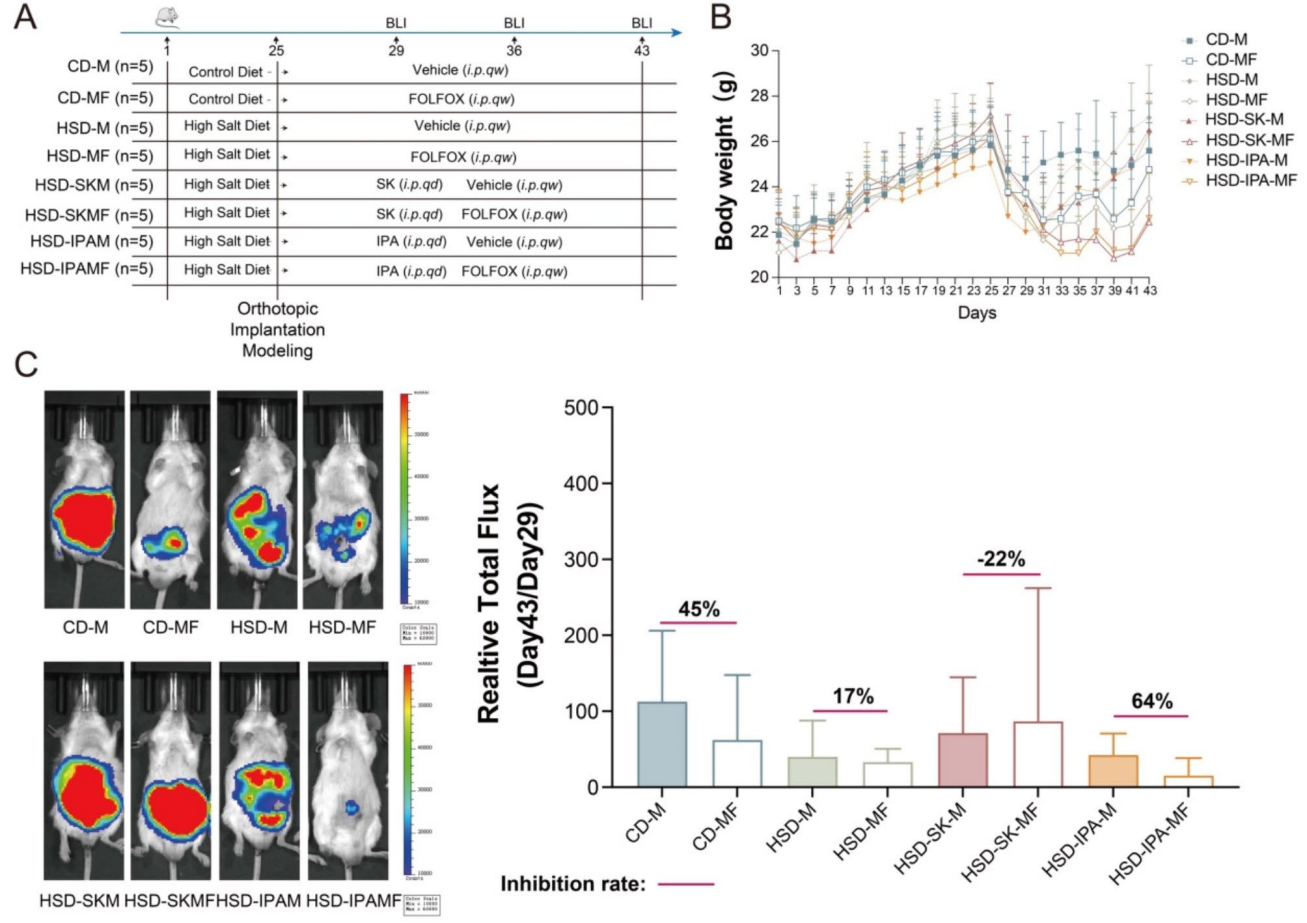
Tryptophan Metabolites Regulate the Efficacy of FOLFOX. (**A**) Schematic diagram of the animal experiment. The high-salt diet groups (HSD-M /HSD-MF/HSD-SKM/HSD-SKMF/HSD-IPAM/HSD-IPAMF) were subjected to high-salt diet intervention throughout the experiment, while the tryptophan metabolite intervention groups (HSD-SKM/HSD-SKMF/HSD-IPAM/HSD-IPAMF) received daily tryptophan administration. (**B**) Changes in mouse body weight throughout the experiment. (**C**) Bioluminescence images and tumor inhibition rates for each group on day 43 (end of the experiment). CD-M: Model group. CD-MF: FOLFOX administration group. HSD-M: High-salt diet group. HSD-MF: High-salt diet intervention combined with FOLFOX administration group. HSD-SKM: SK intervention group. HSD-SKMF: SK intervention combined with FOLFOX administration group. HSD-IPAM: IPA intervention group. HSD-IPAMF: IPA intervention combined with FOLFOX administration group

To investigate the role of SK and IPA in modulating FOLFOX efficacy, we established an orthotopic CRC mouse model with luciferase-labeled CT26 cells (Fig. 8A). As in the previous batch of animal experiments (Fig. 2A), mice were administered either a control diet (CD-M, CD-MF) or a high-salt diet (HSD-M, HSD-MF, HSD-SKM, HSD-SKMF, HSD-IPAM, HSD-IPAMF) throughout the experimental period (Day 1 - Day 43). Consistent with earlier findings, we observed that the high-salt diet did not significantly affect mice body weight (Figs. 2B and 8B), nor did the tryptophan metabolites impact body weight. This indicates that SK and IPA did not adversely affect the overall health of the mice during the experiment. However, FOLFOX treatment did result in weight loss in the CD-MF, HSD-MF, HSD-SKMF and HSD-IPAMF groups.

The tryptophan metabolite intervention groups (HSD-SKM, HSD-SKMF, HSD-IPAM, HSD-IPAMF) were treated daily with tryptophan metabolites SK (20 mg/kg) and IPA (25 mg/kg) starting from the second day after modeling (Day 26). Simultaneously, the treatment groups (CD-MF, HSD-MF, HSD-SKMF, HSD-IPAMF) were intraperitoneally administered FOLFOX or a solvent control (Fig. 8A). By comparing tumor inhibition rates calculated from bioluminescence intensity (Total Flux) measured at the experimental endpoint (Day 43), we found that, consistent with previous results (Fig. 2B), a high-salt diet reduced the efficacy of FOLFOX from 45 to 17% (Fig. 8C).

Interestingly, the two tryptophan metabolites showed distinct effects on the efficacy of FOLFOX. SK reduced the FOLFOX inhibition rate from 17 to 22% under a high-salt diet, aligning with our in vitro observation that SK inhibited FOLFOX efficacy (Figs. 7D and 8C). In contrast, IPA increased the FOLFOX inhibition rate from 17 to 64% under a high-salt diet, which is consistent with our long-term observation (colony formation assay) showing that IPA enhances FOLFOX efficacy in vitro (Fig. 7E), indicating that IPA can potentiate FOLFOX’s effectiveness (Fig. 8C).

In conclusion, by establishing an orthotopic CRC transplantation mouse model, we confirmed that tryptophan metabolites modulate the efficacy of FOLFOX under a high salt diet, with SK inhibiting and IPA promoting its efficacy.

## Discussion

**Fig. 9:**
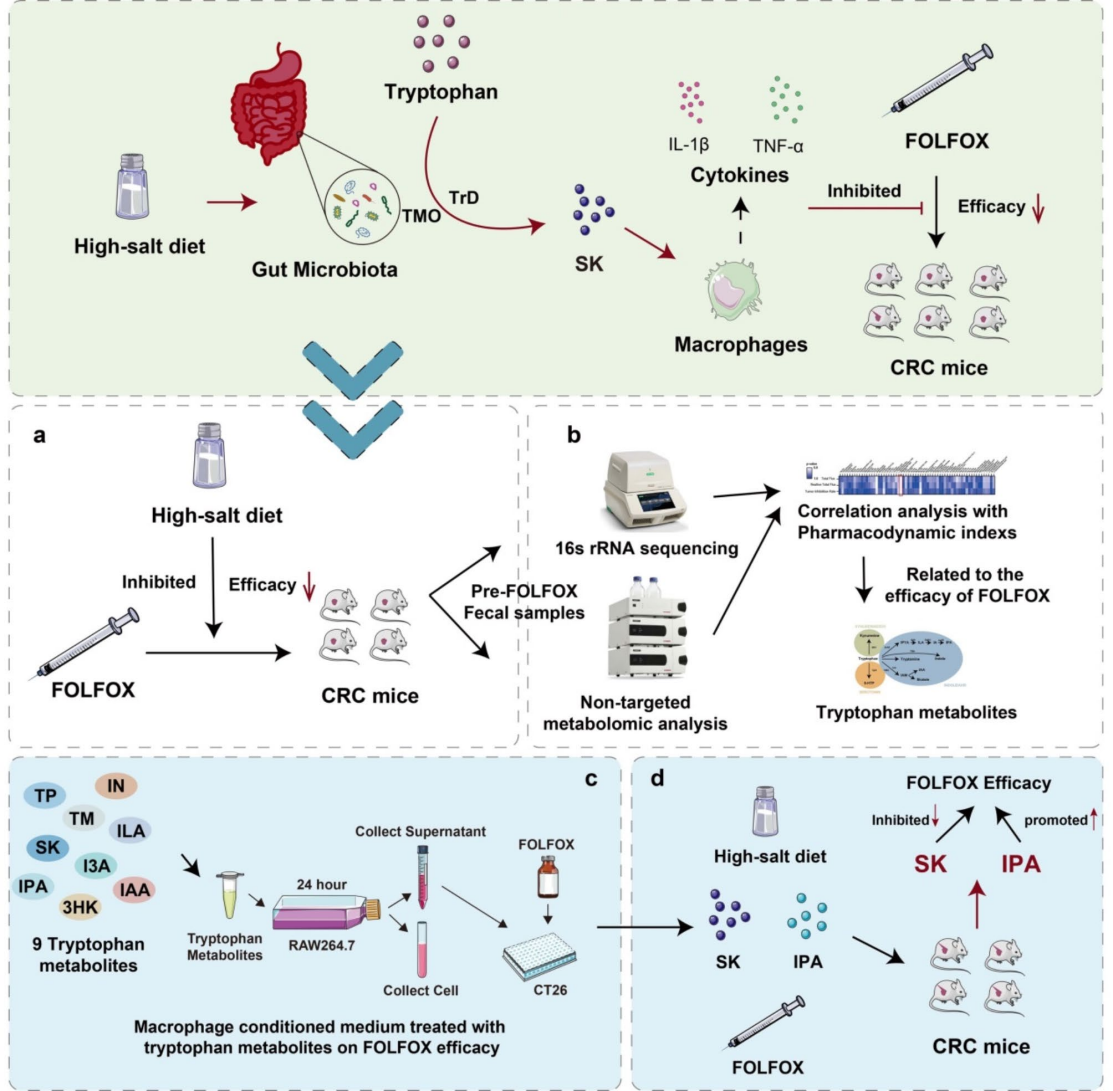
Graphical abstract. The schematic diagram illustrates the process and key findings of our research. Initially, we demonstrated that a high-salt diet inhibits the efficacy of FOLFOX in a mouse model of orthotopic CRC xenografts. Non-targeted metabolomics then identified tryptophan metabolites as been most closely associated with FOLFOX efficacy. Macrophage-conditioned medium containing nine tryptophan metabolites was used to validate their impact on FOLFOX efficacy in vitro. Among these metabolites, SK and IPA were selected for further in vivo validation. Finally, in vivo validation revealed that the tryptophan metabolite SK, when combined with a high-salt diet, suppresses the efficacy of FOLFOX, whereas IPA enhances it

The limited efficacy of FOLFOX has been a major obstacle to anti-CRC chemotherapy. Several studies reported that the expression of *PVT1* gene (Wu et al. 2022) and serum lactate dehydrogenase (LDH) (Koukourakis et al. 2011) might improve the efficacy of FOLFOX against CRC, but there lacks clear elucidation of unsatisfied efficacy of FOLFOX. In this study, we found that high salt diet decreased the efficacy of FOLFOX and changed the profile of gut microbiota and related metabolites. And we confirmed that bacterial tryptophan metabolites IN, SK, I3A antagonized while IPA, IAA synergized FOLFOX efficacy through macrophage mediated immune regulation. Through in vivo experiments, we discovered that tryptophan metabolites can modulate the efficacy of FOLFOX under a high-salt diet, with SK inhibiting the efficacy of FOLFOX and IPA enhancing its therapeutic effect (Fig. 9). Taken together, our study reveals that bacterial tryptophan metabolism participates in the attenuated the anti-CRC effect of FOLFOX induced by high salt diet through tumor immune microenvironment, which gain insight into the mechanism of limited efficacy of FOLFOX, and provide novel target for CRC therapy.

Our results showed that the occurrence and development of CRC affect the composition and structure of gut microbiota as well as the profiles and relative content of gut microbiota metabolites in both mice and humans (Figs. 1 and 3). These observations are consistent with other researches in human (Kong et al. 2023) and mice (Cai et al. 2022; Gou et al. 2023). In recent years, the CRC orthotopic tumor model has become a widely used model for CRC research in mice, as evidenced by recent studies (Shen et al. 2024; Qin et al. 2024). Previous reports have shown alterations in the gut microbiota and metabolites in mice with CRC orthotopic tumors (Qu et al. 2023). Our study observed similar changes in the gut microbiota and metabolites of CRC tumor-bearing mice. This resemblance not only validates the reliability of our experimental findings but also supports the accuracy of our modeling approach. It reinforces the robustness of our research methodology and strengthens the credibility of our findings within the context of CRC mouse model research.

Gut microbiota is known to be involved in chemotherapy efficacy. In our previous studies, we have confirmed that the heterogeneity of intestinal flora and related metabolites are involved in the individualized efficacy of FOLFOX, and gut microbiota *Akkermansia Muciniphila* and *Prevotella* colonization affects the anti-CRC effect of FOLFOX (Hou et al. 2021a, b). However, the underlying mechanism is unclear. As an important bacterial manipulation strategy, special diet has recently been proved to affect cancer development and chemotherapy (Tajan and Vousden 2020). High salt diet is a representative western dietary habit, and has received widespread attention due to its negative impact on health. Based on existing studies, we selected a high concentration (8%) of high-salt diet to explore its impact on anti-tumor treatment. Our findings indicated that a high-salt diet slightly attenuated the development of CRC in orthotopic implantation mouse model, consistent with the study by *Soonjae Hwang* (Hwang et al. 2020). However, a high-salt diet decreased the anti-CRC effect of FOLFOX, as shown by a significantly lower inhibition rate (from 43 to 14%) and altered gut microbiota and metabolites profiles in tumor-bearing mice (Figs. 2 and 4). Interestingly, several recent studies have suggested that a 4% high-salt diet might exert anti-tumor effects or promote anti-tumor efficacy at the animal level (He et al. 2020; Deng et al. 2019; Khandekar et al. 2022; Scirgolea et al. 2024). We speculate that the bidirectional effects of high-salt diets may be due to the concentration of NaCl and the inherent limitations of animal experiments. It would be intriguing to design a range of NaCl doses to investigate the dose-dependent effects of NaCl on CRC mice response to chemotherapy. This approach could provide scientific insights for establishing guidelines on the acceptable daily intake (ADI) of NaCl.

It is known that bacterial metabolites such as SCFAs, bile acids and tryptophan related metabolites are important in gut microbiota function. They are involved in drug response directly or indirectly through immune regulation (Smith et al. 2013; Renga et al. 2022). In this study, we found that high salt diet altered gut bacterial related metabolism. And non-targeted metabolomics data showed that the abundance of bacterial tryptophan metabolite IAA was significantly correlated with the anti-cancer index of FOLFOX, the level of other bacterial tryptophan metabolites (TM, IPA, IAA) were also significantly changed after high salt diet (Fig. 5). Meanwhile, existing studies suggested that bacterial tryptophan metabolism could affect chemotherapy response in various cancers and may be important synergistic targets (Creelan et al. 2013; Li et al. 2014; Tintelnot et al. 2023). Considering the significantly changed level of tryptophan metabolism between the control mice and tumor bearing mice (Suppl. Fig. S5), we further examined the involvement of bacterial derived tryptophan metabolites in high salt attenuated FOLFOX efficacy.

Studies have confirmed that tryptophan metabolites have immune, metabolic, and neuroregulatory functions (Platten et al. 2019). Bacterial tryptophan metabolites such as kynurenine, 5-HTP and indoles are known to affect cancer development and chemotherapy response through immune regulation (Huang et al. 2022a, b). Therefore, we subsequently examined the immune regulatory effect of high salt diet of FOLFOX treatment in mice. Results showed that high salt diet inhibited the infiltration levels of macrophages in CRC as well as in paracancerous tissues (Fig. 6). These data indicate that high salt diet may decrease FOLFOX efficacy through bacterial tryptophan metabolism mediated immune regulation.

Considering the decreased level of macrophages in CRC patients (Suppl. Fig. S10) as well as after high salt diet (Fig. 6). We established an in vitro system of macrophages supplemented with tryptophan metabolites to examine the involvement of immune regulation in FOLFOX efficacy. The important inflammatory factors produced by macrophages has been confirmed to affect tumor immune microenvironment (Zeng et al. 2021; Deng et al. 2022; Wu et al. 2023). Among them, IL-1β and TNF-α contribute to tumor chemotherapy resistance and are associated with poor prognosis of chemotherapy (Mitsunaga et al. 2013). Therefore, we firstly detected the level of IL-1β and TNF-α in macrophages after tryptophan metabolites treatment, and found IN, SK, IPA, and I3A significantly increased the production of them in macrophages while TP, TM and ILA significantly reduced, indicating that bacterial tryptophan metabolism may affect FOLFOX efficacy through immune regulation. Meanwhile, MTT and colony formation assay suggested that IN, SK and I3A significantly antagonized FOLFOX efficacy in CRC indirectly through macrophage conditioned medium (Fig. 7 and Suppl. Fig. S12), while FOLFOX efficacy was not affected when directly combined with tryptophan metabolites (0.85 ≤ Q < 1.15) (Suppl. Fig. S9 and Suppl. Table S6). Interestingly, while inhibited the anti-cancer effect of FOLFOX in short term treatment (MTT), IPA and IAA significantly promoted the effect of FOLFOX after long term exposure (8 days), suggesting the biofunction of IPA and IAA may be transformed during long time interaction with FOLFOX, which deserves in depth exploration. Therefore, we selected SK as antagonistic candidate and IPA as synergistic candidate for in vivo verification. The inhibition rates of FOLFOX and FOLFOX under a high-salt diet were consistent with those observed in the previous batch of experiments (Fig. 2), confirming that the effects of FOLFOX and a high-salt diet remained consistent across both batches of animal models. Our findings revealed that SK and IPA modulated the efficacy of FOLFOX, with SK inhibiting and IPA enhancing the therapeutic effect of FOLFOX.

This study provides an in-depth understanding of the FOLFOX efficacy against CRC, but still has some limitations. We observed altered levels of macrophages in tumor and paracancerous tissues and verified this using an in vitro macrophage conditioned medium. Whether other immune cells, such as cytotoxic T cells, contribute to the decreased FOLFOX efficacy remains unclear and warrants further investigation. Additionally, the functional cytokines responsible for the antagonistic effect observed with tryptophan metabolites in the conditioned medium are yet to be identified. Furthermore, the underlying mechanisms by which SK reduces and IPA enhances the efficacy of FOLFOX require further exploration.

Using CRC orthotopic implantation mice and in vitro models, we demonstrate for the first time that the inhibition of FOLFOX efficacy by a high-salt diet is mediated by gut bacterial tryptophan metabolites, which induce the secretion of macrophage inflammatory cytokines. Specifically, the gut bacterial tryptophan metabolites SK and IPA may play key roles in this process. These findings provide a potential mechanism for the limited anti-CRC efficacy of FOLFOX in clinic. Further elucidating the comprehensive bio-effects of a high-salt diet may provide novel targets for FOLFOX synergistic application in CRC therapy.

## Electronic supplementary material

Below is the link to the electronic supplementary material.

Supplementary Material 1

## Data Availability

Data will be made available on request. The sequence data that support the findings of this study are openly available in the Sequence Read Archive (SRA) under the accession number PRJNA1047082 (Clinical samples) and PRJNA1047661 (Animal samples).
