# Supplementary material for: High-salt diet decreases FOLFOX efficacy via gut bacterial tryptophan metabolism in colorectal cancer
Source: Mol Med. 2025 Feb 19;31:66. doi: 10.1186/s10020-025-01122-8 (PMC11841010; doi:10.1186/s10020-025-01122-8)
Supplement: Supplementary file 1 — Supplementary Material 1 [file 10020_2025_1122_MOESM1_ESM.docx]

**Supplementary materials for**

**High-salt diet decrease FOLFOX efficacy via gut bacterial tryptophan metabolism in colorectal cancer**

**1. Supplementary Material and Methods**

***1.1 Tryptophan metabolites, tryptophan metabolite conditioned medium, and FOLFOX dosage of MTT***

C: Control conditioned medium; TP: Tryptophan treatment conditioned medium, 50 μM; TM: Tryptamine treatment conditioned medium, 5 μM; IN: Indole treatment conditioned medium, 500 μM; SK: Skatole treatment conditioned medium, 100 μM; ILA: Indole-3-Lactic Acid treatment conditioned medium, 800 μM; IPA: 3-Indole propionic acid treatment conditioned medium, 500 μM; I3A: Indole-3-carboxaldehyde treatment conditioned medium, 100 μM; 3HK: Kynurenine treatment conditioned medium, 500 μM; IAA: 3-Indoleacetic acid treatment conditioned medium­­­­, 1000 μM.

Cell growth inhibition was assessed using the MTT assay. Briefly, cells were seeded in 96-well plates and allowed to attach overnight. After 72 hours of drug incubation at various concentrations (37°C), MTT reagent (5 mg/mL, 20 μL/well) was added to each well and incubated for an additional 4 hours. Then the supernatant was discarded, and the cell pellets were dissolved in 200 uL DMSO. Absorbance was measured using a Microplate Reader (Thermo Fisher Scientific, Massachusetts, USA) at a wavelength of 570 nm. All experiments were performed in triplicate.

***1.2 Tryptophan metabolites, tryptophan metabolite conditioned medium, and FOLFOX dosage of*** ***Colony formation assay***

C: Control medium C-C: Control conditioned medium; IN-C: Indole treatment conditioned medium, 500 μM; IN-M: Indole medium, 500 μM; SK-C: Skatole treatment conditioned medium, 20 μM; SK-M: Skatole medium, 20 μM; IPA-C: 3-Indole propionic acid treatment conditioned medium, 100 μM; IPA-M: 3-Indole propionic acid medium, 100 μM; I3A-C: Indole-3-carboxaldehyde treatment conditioned medium, 20 μM; I3A-M: Indole-3-carboxaldehyde medium, 20 μM; IAA-C: 3-Indoleacetic acid treatment conditioned medium, 1000 μM; IAA-M: 3-Indoleacetic acid medium, 1000 μM.

For the colony formation assay, CRC cells (800 cells/well) were seeded in 6-well plates and allowed to adhere overnight. Subsequently, the cells were treated with FOLFOX/tryptophan metabolite with or without conditioned medium. The medium was refreshed every 2 days, and the cells were cultured for approximately 8 days. Afterwards, colonies were fixed with 4% paraformaldehyde (PFA) and stained with 0.5% (w/v) crystal violet. The colonies were then imaged and counted.

**2. Supplementary figures**


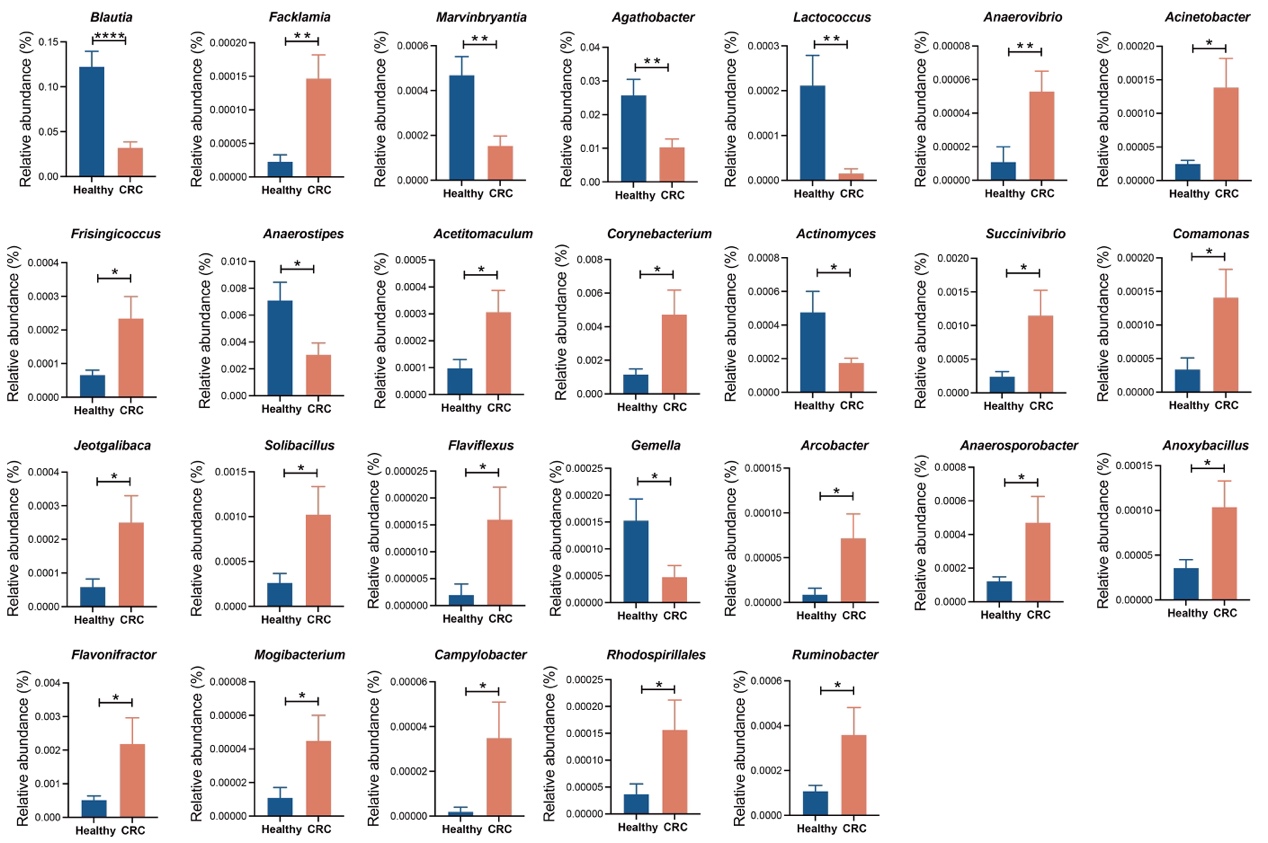


**Figure.S1 Relative abundance of 26 differential gut bacteria in fecal samples between Healthy adults (n=9) and CRC patients (n=6).** * *p* <0.05, ** *p* <0.01, **** *p* <0.0001.


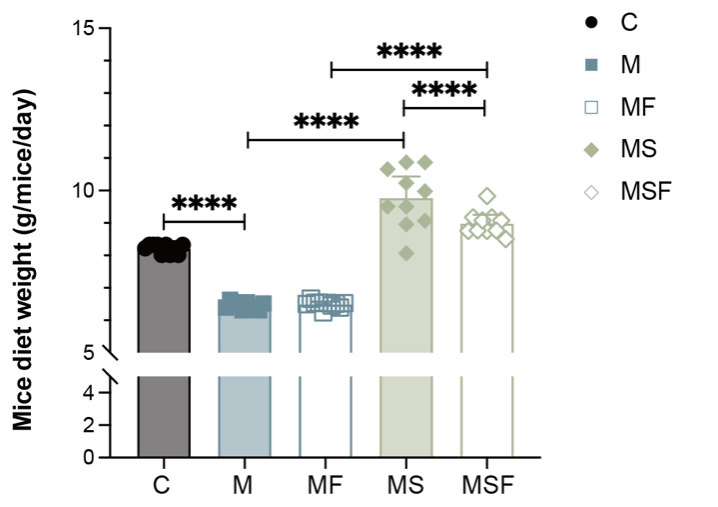


**Figure.S2 Increased feed consumption in high salt diet group mice.** Control group (C), Model group (M), FOLFOX group (MF), High salt diet group (MS), and High salt diet combined with FOLFOX group (MSF). Calculation of the daily feed consumption of each mouse. **** *p* <0.0001.

**
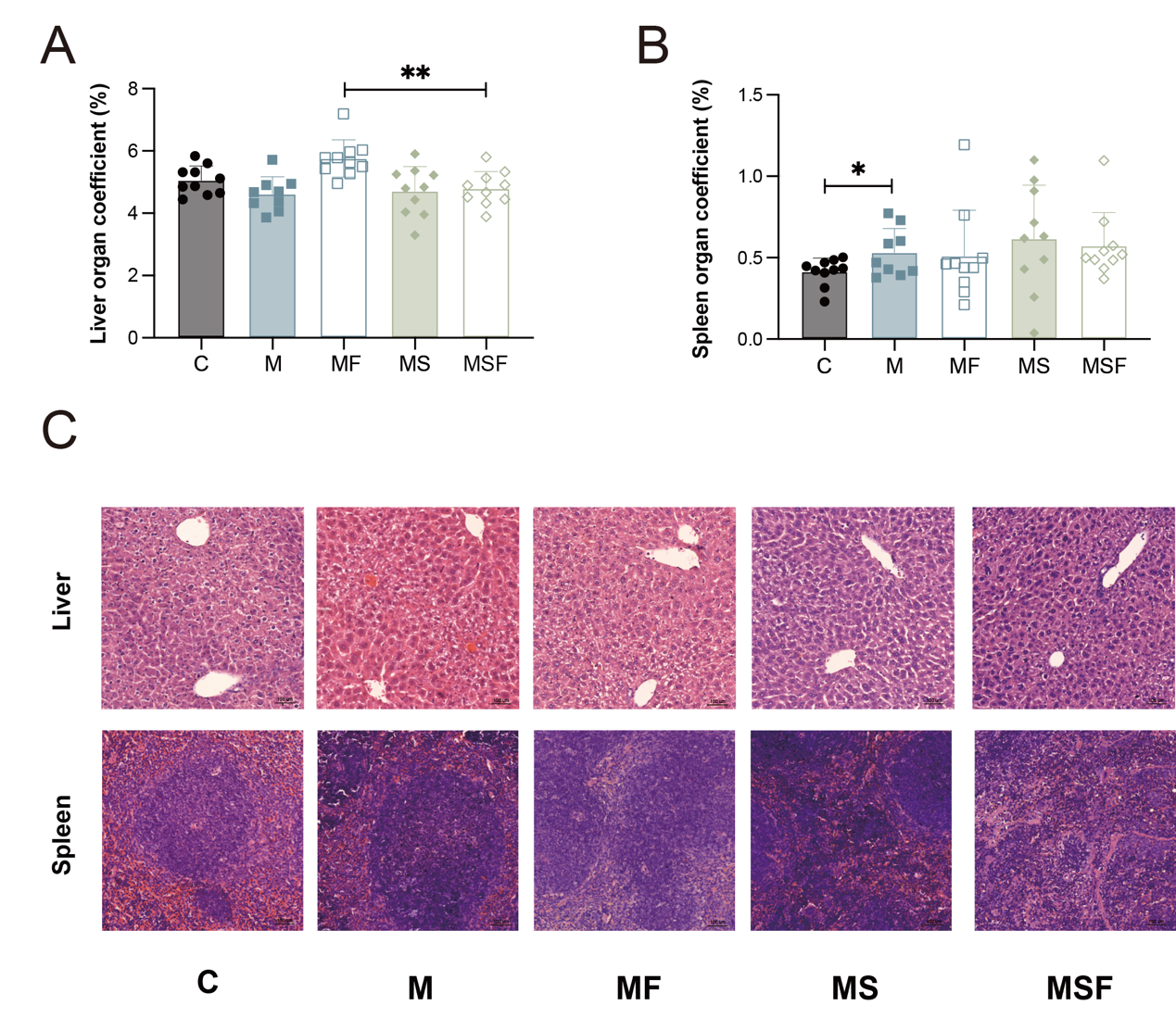
**

**Figure.S3 High salt diet and FOLFOX have no toxic effects on the liver and spleen.** (A) Liver organ coefficients of each group of mice. (B) Spleen organ coefficients of each group of mice. (C) HE staining of liver and spleen from mice in each group. * *p* <0.05, ** *p* <0.01.

**
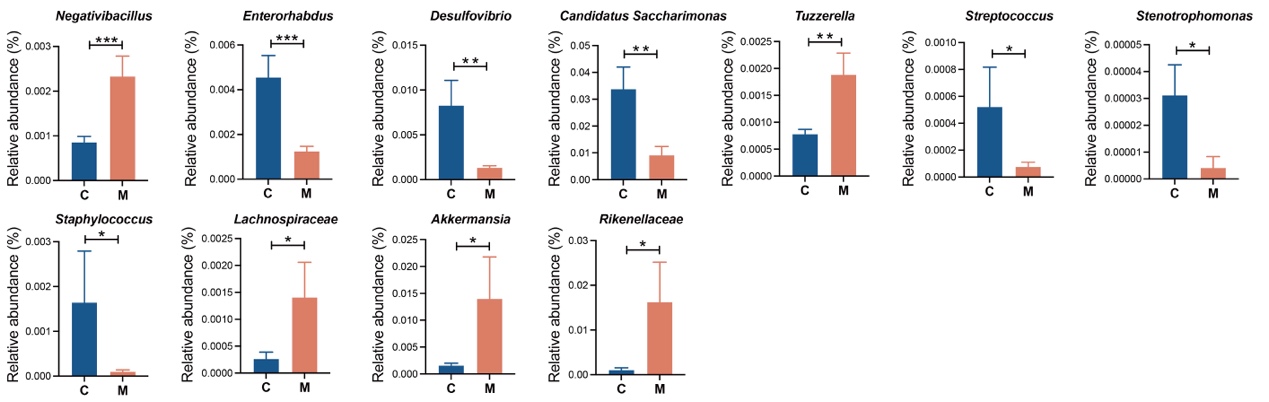
**

**Figure.S4 Relative abundance of 11 differential gut bacteria in fecal samples between Control (C) and Model (M) mice.** * *p* <0.05, ** *p* <0.01, *** *p* <0.001.

**
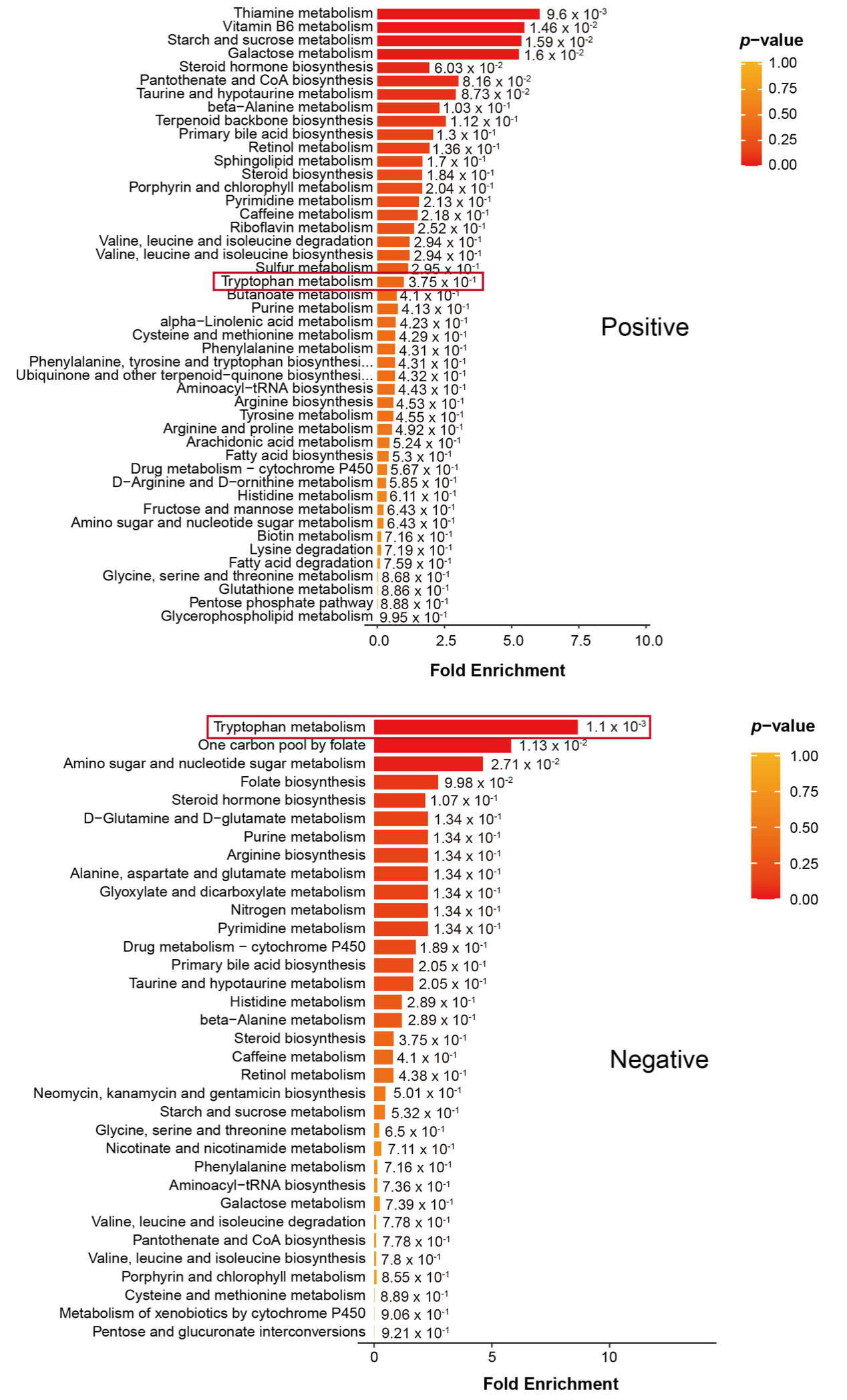
**

**Figure.S5 MESA pathway enrichment analysis between Control and Model mice.** Level of tryptophan metabolism was significantly different between Control and Model mice. Positive, positive ion mode. Negative, negative ion mode.

**
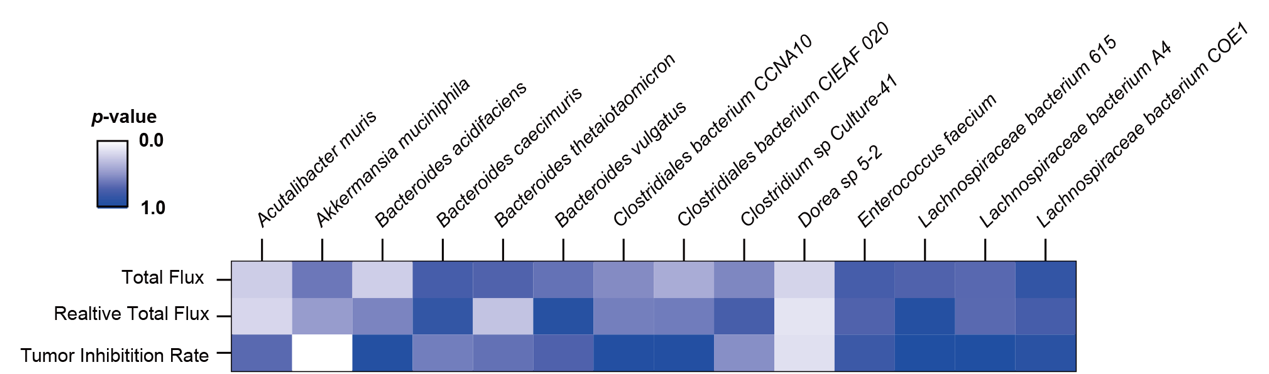
**

**Figure.S6 The level of differential bacteria is not significantly correlated with the efficacy of FOLFOX in CRC.** Spearman correlation analysis was conducted between the differential bacteria of all CRC bearing mice (n=40) and the pharmacodynamic evaluation index (Total flux, relative total flux and tumor inhibition rate).

**
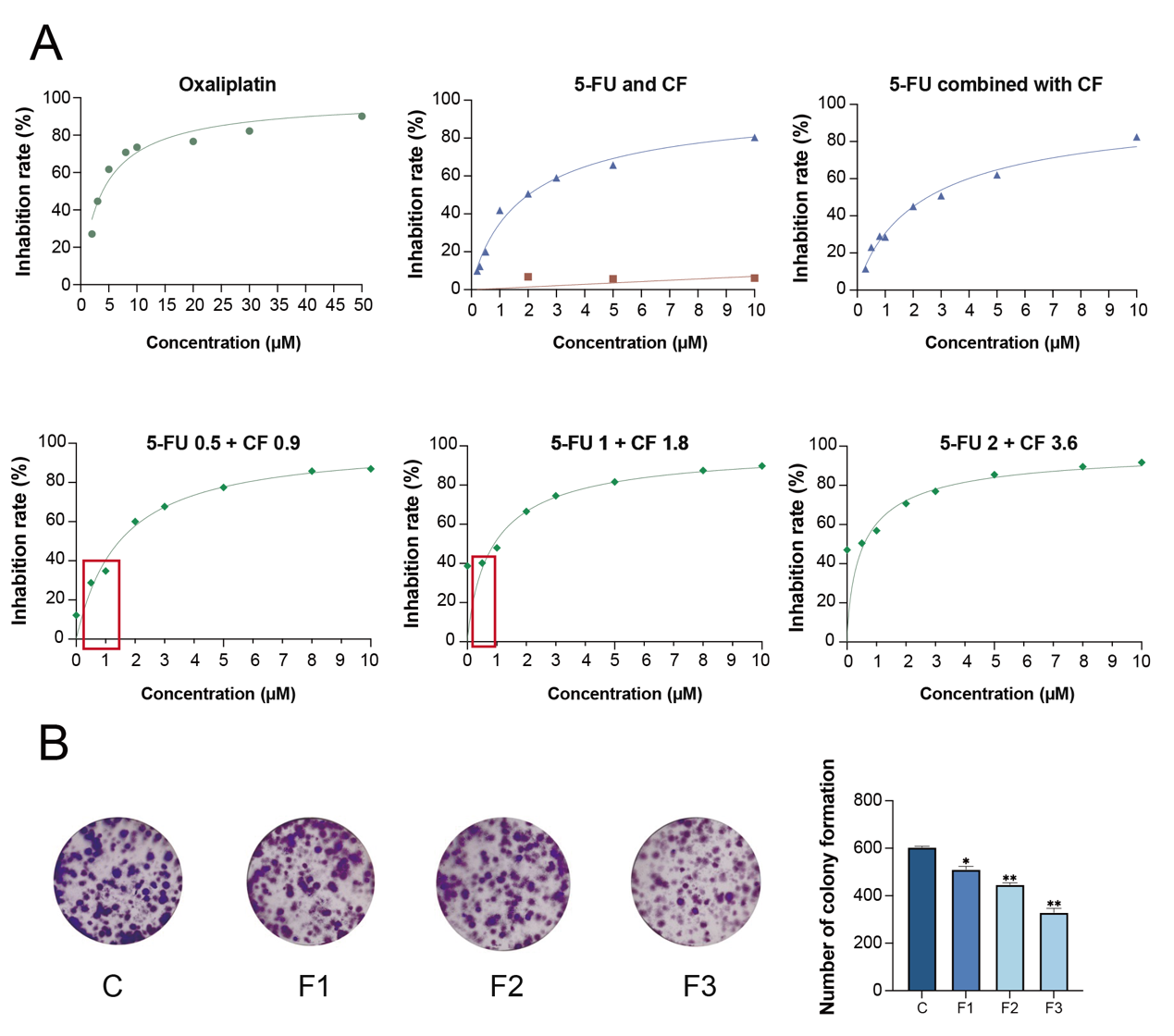
**

**Figure.S7 MTT and colony formation assay for FOLFOX concentration determination.** (A) MTT for FOLFOX concentration determination. 5-FU, Fluorouracil; CF, Calcium folinate. (B) Colony formation assay for FOLFOX concentration determination. * *p* <0.05, ** *p* <0.01.

**
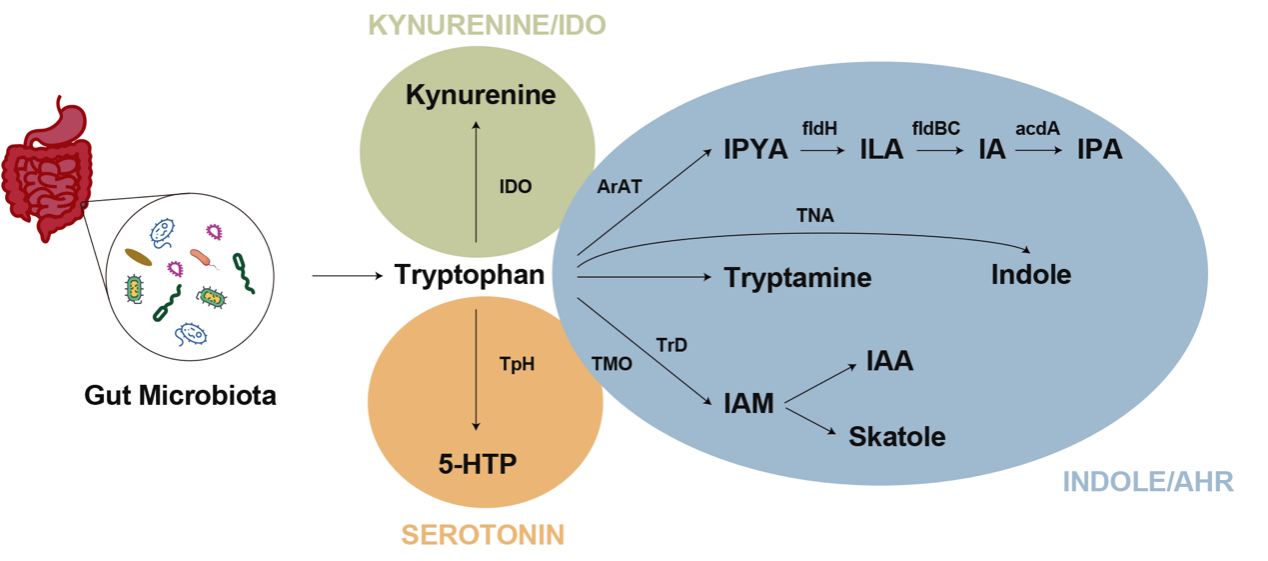
**

**Figure.S8 Tryptophan metabolic pathway of intestinal microbiota.** IPYA: Indole-3-Pyruvate, ILA: Indole-3-Lactic Acid, IA: Indole Acrylic Acid, IPA: 3-Indole propionic acid, IAM: Indole-3-Acetamide, IAA: 3-Indoleacetic acid, 5-HTP: 5-Hydroxytryptophan, IDO: Indoleamine 2,3-Dioxygenase, ArAT: Aromatic amino acid aminotransferase, fldH: phenyllactate dehydrogenase, fldBC: phenyllactate dehydratase, acdA: acyl-CoA dehydrogenase, TNA: Tryptophanase, TMO: Tryptophan 2-Monooxygenase, TrD: Tryptophan Decarboxylase, TpH: Tryptophan Hydroxylase.

**
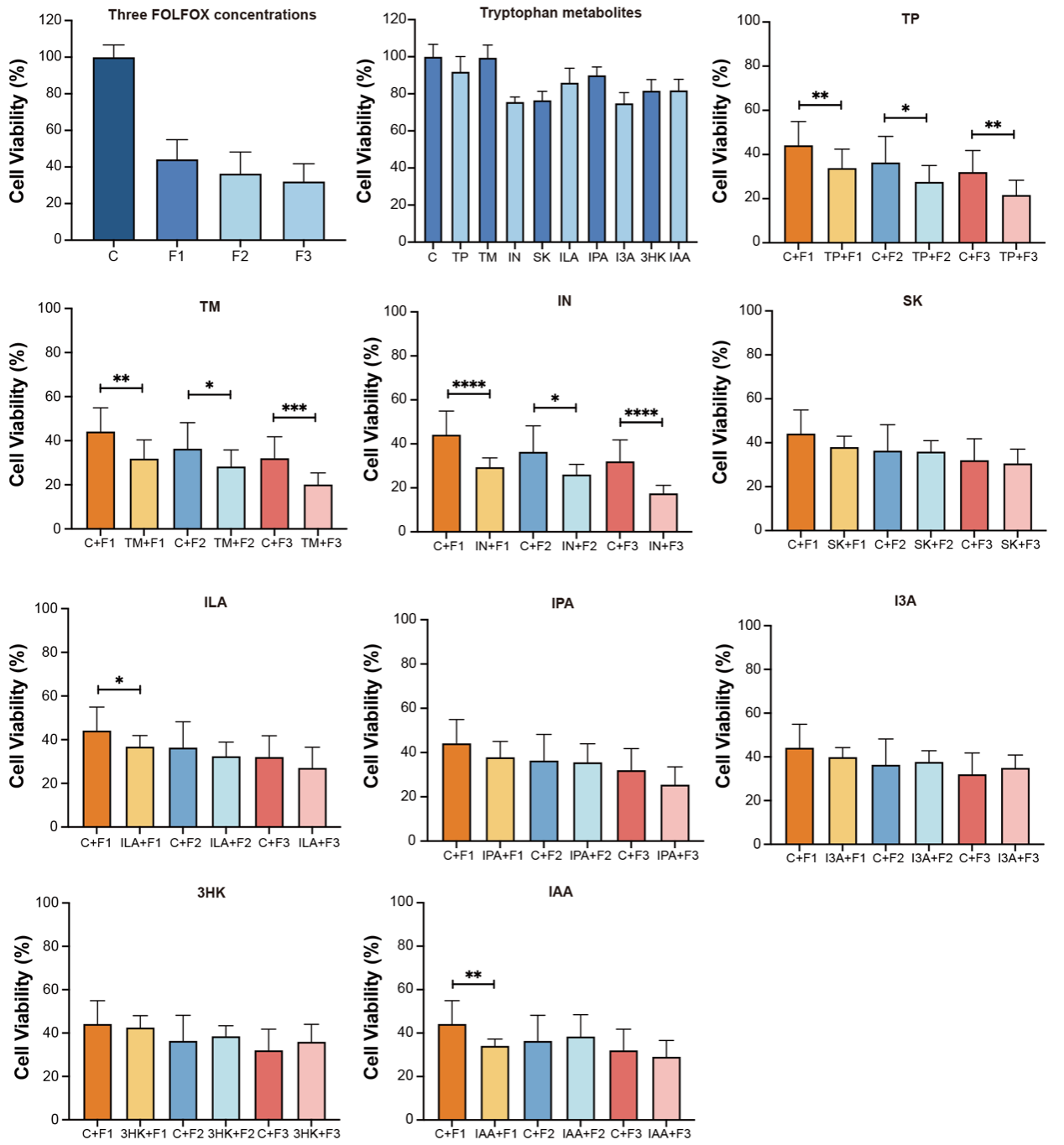
**

**Figure.S9 Effect of tryptophan metabolites on the** **anti-proliferation effect of FOLFOX *in vitro*.** C: Control, TP: Tryptophan, 50 μM; TM: Tryptamine, 5 μM; IN: Indole, 500 μM; SK: Skatole, 100 μM; ILA: Indole-3-Lactic Acid, 800 μM; IPA: 3-Indole propionic acid, 500 μM; I3A: Indole-3-carboxaldehyde, 100 μM; 3HK: Kynurenine, 500 μM; IAA: 3-Indoleacetic acid­­­­, 1000 μM. * *p* <0.05, ** *p* <0.01, *** *p* <0.001, **** *p* <0.0001.


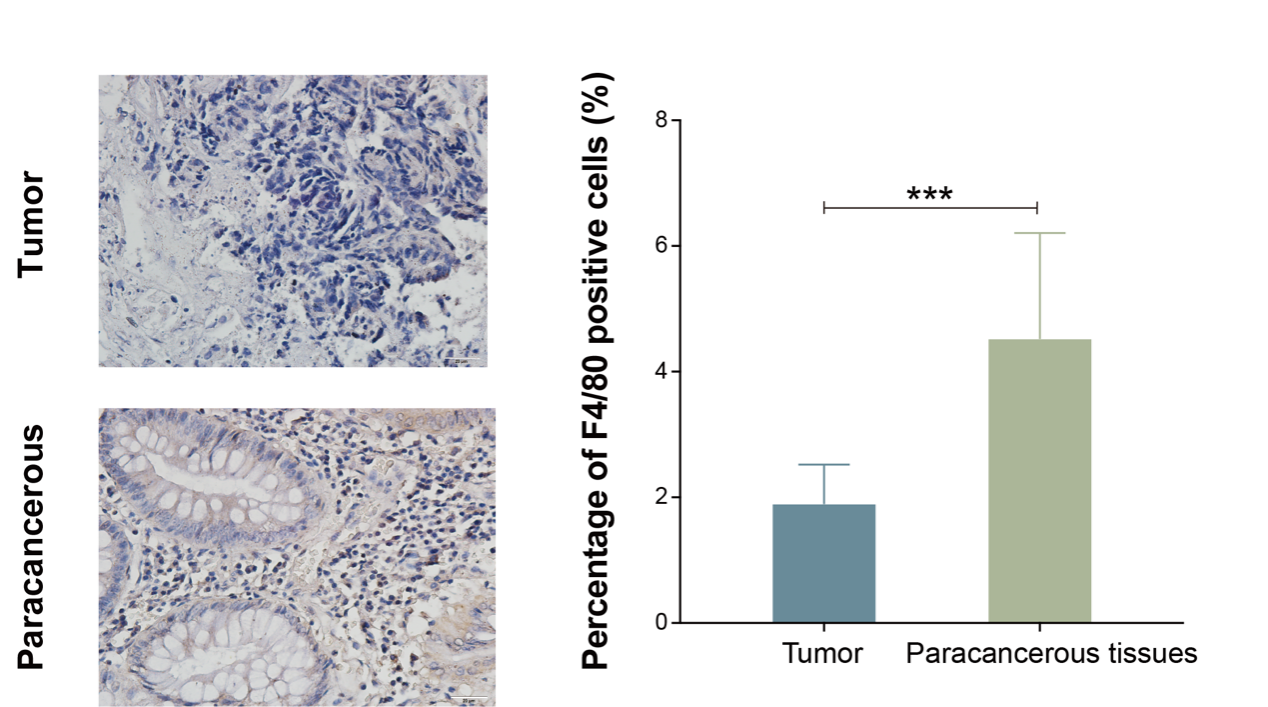


**Figure.S10 Immunohistochemistry (F4/80) of CRC patients in tumor and paracancerous tissues.** *** *p* <0.001.

**
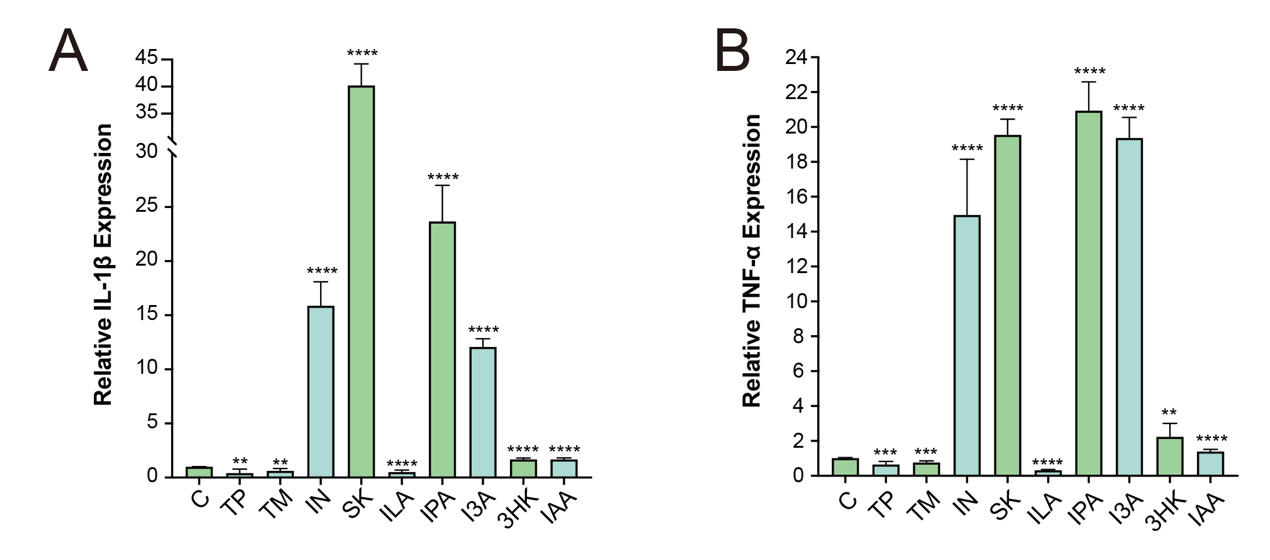
**

**Figure.S11 Tryptophan metabolite significantly increased the expression of IL-1β (A) and TNF-α (B) in macrophages.** C: Control, TP: Tryptophan, TM: Tryptamine, IN: Indole, SK: Skatole, ILA: Indole-3-Lactic Acid, IPA: 3-Indole propionic acid, I3A: Indole-3-carbaldehide, 3HK: Kynurenine, IAA: 3-Indoleacetic acid. ** *p* <0.01, *** *p* <0.001, **** *p* <0.0001.


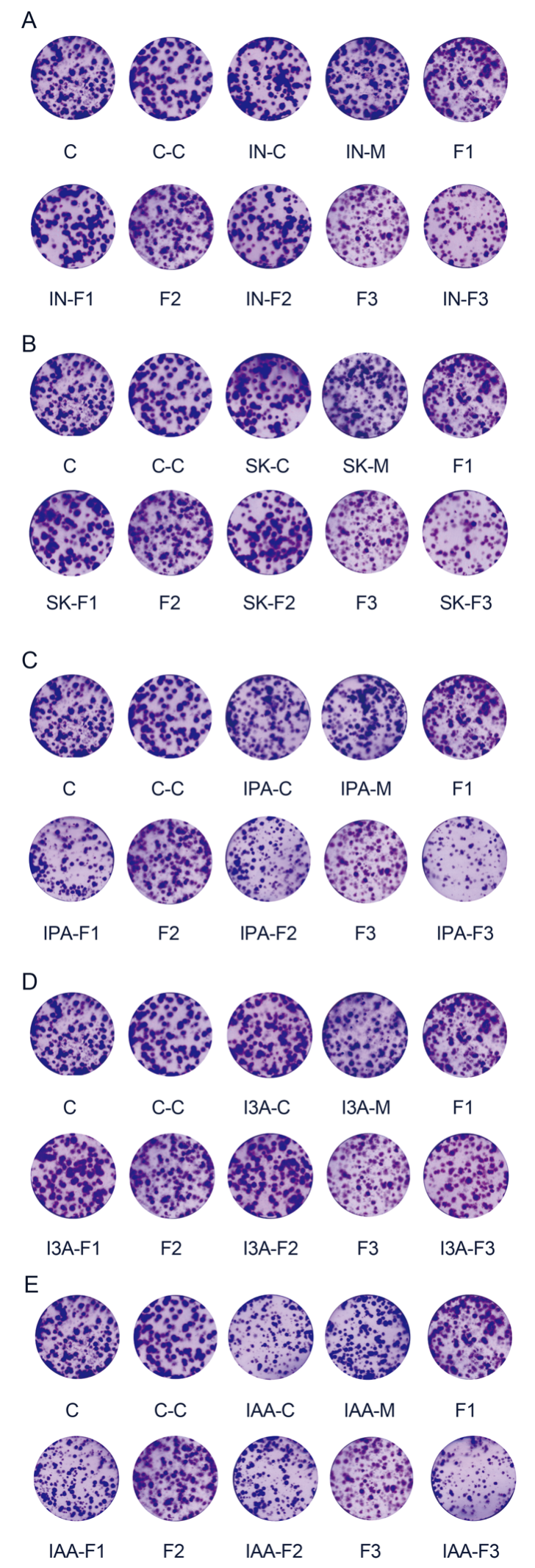


**Figure.S12 Representative images of colony formation assay.** (A) IN. (B) SK. (C) I3A. (D) IPA. (E) IAA. C: Control medium C-C: Control conditioned medium; IN-C: Indole treatment conditioned medium, 500 μM; IN-M: Indole medium, 500 μM; SK-C: Skatole treatment conditioned medium, 20 μM; SK-M: Skatole medium, 20 μM; IPA-C: 3-Indole propionic acid treatment conditioned medium, 100 μM; IPA-M: 3-Indole propionic acid medium, 100 μM; I3A-C: Indole-3-carboxaldehyde treatment conditioned medium, 20 μM; I3A-M: Indole-3-carboxaldehyde medium, 20 μM; IAA-C: 3-Indoleacetic acid treatment conditioned medium, 1000 μM; IAA-M: 3-Indoleacetic acid medium, 1000 μM.

**3. Supplementary tables**

**Table S1. Information table of stool samples collected from 9** **healthy volunteers and 6 CRC patients**. Among the 6 CRC patients, the lesion sites included 3 cases of right-sided colon cancer, 2 cases of left-sided colon cancer, and 1 case of colon cancer with an undetermined site.

|  | **CRC patients**  **（n=6）** | **Healthy volunteer**  **（n=9）** |
| --- | --- | --- |
| Sex |  |  |
| Male | 4 | 5 |
| Female | 2 | 4 |
| Age |  |  |
| < 60 | 2 | 4 |
| ≥ 60 | 4 | 5 |

**Table S2. Information table of tumor tissues and paired paracancerous tissues obtained from 10 CRC patients**.

|  | **CRC patients**  **（n=10）** |
| --- | --- |
| Sex |  |
| Male | 4 |
| Female | 6 |
| Age |  |
| < 60 | 3 |
| ≥ 60 | 7 |
| Subsite |  |
| Left-sided colon | 2 |
| Right-sided colon | 1 |
| Rectum | 7 |

**Table S3.** Differential metabolites in fecal samples between CRC patients and healthy adults. Only features with VIP > 1 and Fold Change > 2 or < 0.5 were listed. Metabolites marked with an asterisk (*) are of microbial origin.

| **No.** | **Metabolites** | **VIP** | **Fold Change** |
| --- | --- | --- | --- |
| 1 | Hexadecanedioic acid | 1.18 | 37.496 |
| 2 | Phe phe | 1.46 | 9.229 |
| 3 | N-formylkynurenine | 1.29 | 8.618 |
| 4 | 3-Methyloxindole | 1.13 | 7.707 |
| 5 | Hexylamine* | 1.59 | 7.418 |
| 6 | Indole-3-carboxylic acid* | 2.20 | 7.392 |
| 7 | P-Aminobenzoate* | 1.26 | 7.340 |
| 8 | Phthalic acid | 1.29 | 4.421 |
| 9 | 1,5-Diaminopentane* | 1.43 | 4.409 |
| 10 | Isoallolithocholic acid* | 1.28 | 4.062 |
| 11 | 2-Aminooctanoic acid | 1.13 | 3.248 |
| 12 | 3-Methyl-1-butylamine | 2.28 | 3.143 |
| 13 | Octadecanedioate (C18) | 2.22 | 3.127 |
| 14 | L-Cystine | 1.52 | 3.092 |
| 15 | γ-Glu-Met | 1.46 | 2.982 |
| 16 | Xanthurenic Acid | 1.16 | 2.787 |
| 17 | Gly-Val | 1.65 | 2.743 |
| 18 | N-Acetyl-L-Tyrosine | 1.71 | 2.742 |
| 19 | Putrescine* | 1.14 | 2.727 |
| 20 | PYRROLIDINE | 1.07 | 2.695 |
| 21 | Phe Leu | 1.43 | 2.645 |
| 22 | γ-Glu-Phe | 2.02 | 2.641 |
| 23 | γ-glutamylalanine | 1.07 | 2.633 |
| 24 | β-indole-3-acetic acid | 1.18 | 2.474 |
| 25 | Pyridoxamine dichlorohydrate | 2.41 | 2.404 |
| 26 | 3-Phenylpyruvic Acid* | 1.02 | 2.378 |
| 27 | 12-ketolithocholic acid* | 1.30 | 2.299 |
| 28 | L-Asparagine | 1.45 | 2.191 |
| 29 | Isolithocholic acid* | 1.03 | 2.126 |
| 30 | Adenine | 1.84 | 2.102 |
| 31 | Xanthosine | 1.28 | 0.485 |
| 32 | Inosine | 1.33 | 0.482 |
| 33 | Glycohyocholic acid* | 1.19 | 0.478 |
| 34 | disodium D-fructose-6-phosphate | 1.07 | 0.468 |
| 35 | 2'-O-methylcytidine | 1.07 | 0.466 |
| 36 | Trimethylamine N-Oxide* | 1.07 | 0.458 |
| 37 | Salicylic acid | 1.24 | 0.451 |
| 38 | 3-N-Methyl-L-Histidine | 1.15 | 0.429 |
| 39 | 5-Methyluridine | 1.59 | 0.426 |
| 40 | Ursocholic acid* | 1.28 | 0.409 |
| 41 | Xylonate | 1.33 | 0.393 |
| 42 | 2-Methyllactic acid* | 1.46 | 0.369 |
| 43 | Taurochenodeoxycholic acid | 1.26 | 0.351 |
| 44 | Taurodeoxycholic acid | 1.28 | 0.344 |
| 45 | 7-ketolithocholic acid | 1.14 | 0.337 |
| 46 | 3-hydroxymethylglutaric acid | 1.54 | 0.284 |
| 47 | dTMP | 1.23 | 0.282 |
| 48 | Trehalose | 1.10 | 0.272 |
| 49 | Anserine | 1.24 | 0.269 |
| 50 | Cytidine | 1.75 | 0.253 |
| 51 | 7-methylxanthine | 1.44 | 0.244 |
| 52 | 7-Ketodeoxycholic acid* | 1.37 | 0.220 |
| 53 | Cholic acid | 1.50 | 0.215 |
| 54 | Guanosine | 1.79 | 0.210 |
| 55 | Tauroursodeoxycholic acid | 1.05 | 0.208 |
| 56 | Uridine | 2.31 | 0.203 |
| 57 | 3β-Cholic Acid | 1.25 | 0.203 |
| 58 | Dihydrothymine | 1.37 | 0.191 |
| 59 | Caffeicacid | 1.28 | 0.178 |
| 60 | 2-Methoxybenzoic acid | 1.88 | 0.170 |
| 61 | Cyclohexylamine | 1.49 | 0.169 |
| 62 | 5-methylcytidine | 1.88 | 0.160 |
| 63 | 4-hydroxyhippuric acid | 1.92 | 0.149 |
| 64 | D-Erythrose 4-phosphate | 1.26 | 0.148 |
| 65 | dUMP | 2.23 | 0.145 |
| 66 | Dihydrocaffeic acid | 1.21 | 0.143 |
| 67 | Tryptamine* | 1.15 | 0.142 |
| 68 | Trans-4-Hydroxy-L-Proline | 1.20 | 0.138 |
| 69 | Chenodeoxycholic acid | 1.43 | 0.120 |
| 70 | Gallicacid | 1.42 | 0.106 |
| 71 | Taurocholic acid | 1.21 | 0.095 |
| 72 | (3,4-Dimethoxyphenyl) Acetic Acid | 1.62 | 0.086 |
| 73 | Citric acid | 2.34 | 0.075 |
| 74 | Creatine | 1.59 | 0.059 |
| 75 | Arachidonoyl ethanolamide | 1.03 | 0.056 |
| 76 | Creatinine | 1.87 | 0.046 |
| 77 | 3-Oxocholic acid* | 1.18 | 0.040 |
| 78 | 1-methyladenosine | 1.62 | 0.026 |
| 79 | N6-methyladenosine | 1.58 | 0.007 |
| 80 | Cyclamic acid | 1.92 | 0.005 |

**Table S4.** Differential metabolites in fecal samples between control mice and CRC bearing mice. Only features with VIP > 1, *p* < 0.05 and Fold Change > 2 or < 0.5 were listed.

| **No.** | **Metabolites** | **VIP** | *p* value | **Fold Change** |
| --- | --- | --- | --- | --- |
| 1 | 11b-Hydroxyprogesterone | 1.74 | 0.038 | 28.22 |
| 2 | Ser-Ala | 2.10 | 0.018 | 23.60 |
| 3 | l-Alanyl-l-glutamine | 2.16 | 0.014 | 23.42 |
| 4 | 5-Phenylvaleric acid | 1.62 | 0.007 | 17.93 |
| 5 | 11-Epimisoprostol | 1.61 | 0.038 | 10.54 |
| 6 | Chlorobiocic acid | 1.07 | 0.030 | 8.87 |
| 7 | 6-Hydroxypentadecanedioic acid | 1.55 | 0.040 | 6.14 |
| 8 | Tauroursodeoxycholic acid | 1.84 | 0.001 | 6.01 |
| 9 | Vobtusine | 2.62 | 0.014 | 6.01 |
| 10 | Val-Ala | 2.40 | 0.010 | 5.82 |
| 11 | 6alpha-Hydroxyteasterone | 2.46 | 0.036 | 5.16 |
| 12 | Arg-Trp | 1.75 | 0.013 | 5.12 |
| 13 | 4-Methoxyphenylacetic acid | 2.10 | 0.006 | 4.48 |
| 14 | Carnitine C20:5 | 2.84 | <0.001 | 4.14 |
| 15 | Asn-Phe | 1.53 | 0.036 | 3.79 |
| 16 | Ile-Glu | 1.40 | 0.023 | 3.70 |
| 17 | Citrulline | 1.77 | 0.023 | 3.34 |
| 18 | Dienestrol | 1.87 | 0.012 | 3.29 |
| 19 | Monobutyl phthalate | 1.74 | 0.008 | 3.15 |
| 20 | Carnitine C7:0 | 1.64 | 0.011 | 3.06 |
| 21 | 1-Stearoyl-sn-glycerol | 2.96 | <0.001 | 2.91 |
| 22 | Biuret | 2.17 | 0.001 | 2.79 |
| 23 | N-Acetyl-L-arginine | 1.00 | 0.023 | 2.75 |
| 24 | Tyr-glu | 1.31 | 0.009 | 2.67 |
| 25 | Glu-Arg | 1.77 | 0.034 | 2.59 |
| 26 | Vitamin K | 2.05 | 0.012 | 2.57 |
| 27 | Leu-Glu | 1.57 | 0.002 | 2.56 |
| 28 | Staphyloxanthin | 1.76 | 0.028 | 2.49 |
| 29 | 1-Vaccenoyl-glycerol | 2.92 | <0.001 | 2.49 |
| 30 | Ethyl palmitoleate | 2.89 | <0.001 | 2.44 |
| 31 | Phenolphthalein | 1.51 | 0.011 | 2.36 |
| 32 | N-Acetylmannosamine | 1.71 | 0.042 | 2.30 |
| 33 | 11-Ketorockogenin acetate | 1.95 | 0.016 | 2.28 |
| 34 | Dehydrocholic acid | 1.07 | 0.043 | 2.18 |
| 35 | Asp-Val | 1.42 | 0.018 | 2.16 |
| 36 | 3-Hydroxydodecanoic acid | 1.35 | 0.029 | 2.14 |
| 37 | L-Tryptophan-L-glutamine | 1.29 | 0.019 | 2.11 |
| 38 | Trp-Arg | 1.92 | 0.017 | 2.07 |
| 39 | 19-Oxoandrostenedione | 2.78 | <0.001 | 2.04 |
| 40 | 2-Oxovaleric acid | 2.28 | 0.001 | 2.02 |
| 41 | Isoleucylaspartic acid | 2.12 | 0.017 | 1.98 |
| 42 | His-Arg | 1.54 | 0.023 | 1.96 |
| 43 | Ile-Arg | 1.62 | 0.003 | 1.92 |
| 44 | Glycohyodeoxycholic acid | 1.61 | 0.028 | 1.90 |
| 45 | Threonyllysine | 1.30 | 0.028 | 1.86 |
| 46 | 4-Acetamidobutanoic acid | 1.93 | 0.026 | 1.86 |
| 47 | Alanylproline | 1.01 | 0.043 | 1.84 |
| 48 | 4-Hydroxybutyric acid | 1.95 | 0.042 | 1.82 |
| 49 | Methyl cholate | 1.75 | 0.006 | 1.82 |
| 50 | Pentylone | 1.69 | 0.016 | 1.82 |
| 51 | Nicotinic acid | 2.30 | 0.007 | 1.78 |
| 52 | 6-Hydroxynicotinic acid | 2.18 | 0.008 | 1.77 |
| 53 | 1-O-Hexadecyl-2-acetyl-sn-glycerol | 2.32 | 0.004 | 1.77 |
| 54 | Trp-Leu | 1.39 | 0.039 | 1.76 |
| 55 | Glycylproline | 1.31 | 0.028 | 1.75 |
| 56 | 3-Hydroxy-5-cholestenoic acid | 1.58 | 0.042 | 1.73 |
| 57 | Picolinic acid | 2.15 | 0.011 | 1.70 |
| 58 | 11-Ketoetiocholanolone | 2.05 | 0.019 | 1.69 |
| 59 | 5-Deoxystrigol | 2.46 | 0.001 | 1.66 |
| 60 | Carnitine C19:0 | 1.47 | 0.044 | 1.64 |
| 61 | Isoleucylproline | 1.67 | 0.019 | 1.64 |
| 62 | N1-Acetylspermidine | 1.59 | 0.034 | 1.63 |
| 63 | Nicotinamide N-oxide | 1.67 | 0.021 | 1.62 |
| 64 | D-piperidine acid | 1.84 | 0.019 | 1.61 |
| 65 | Poststerone | 1.48 | 0.042 | 1.55 |
| 66 | Calcitriol | 1.58 | 0.036 | 1.53 |
| 67 | Adenosine | 1.76 | 0.037 | 1.48 |
| 68 | Glycocholic acid | 1.63 | 0.044 | 1.48 |
| 69 | N6-Acetyl-L-lysine | 1.19 | 0.048 | 1.47 |
| 70 | 7-Ketocholesterol | 2.50 | 0.002 | 1.46 |
| 71 | 3-Hydroxydecanoic acid | 2.08 | 0.021 | 1.44 |
| 72 | Ergosterol | 2.27 | 0.004 | 1.44 |
| 73 | 11-Aminoundecanoic acid | 2.02 | 0.016 | 1.44 |
| 74 | δ-Valerolactam | 2.19 | 0.004 | 1.40 |
| 75 | Noladin ether | 1.98 | 0.029 | 1.30 |
| 76 | Toluene | 2.59 | <0.001 | 1.27 |
| 77 | Methy Indole-3-Acetate | 1.65 | 0.036 | 0.94 |
| 78 | 2,2-Dimethylpentanoic acid | 1.40 | 0.044 | 0.92 |
| 79 | Tyrosylleucine | 1.86 | 0.015 | 0.90 |
| 80 | Valproic acid | 1.77 | 0.028 | 0.89 |
| 81 | Tretinoin | 2.23 | 0.005 | 0.81 |
| 82 | Vanillin | 1.98 | 0.020 | 0.76 |
| 83 | gamma-Linolenic Acid | 2.35 | 0.006 | 0.74 |
| 84 | 9-Octadecynoic acid | 1.84 | 0.040 | 0.74 |
| 85 | Linoelaidic acid | 1.93 | 0.031 | 0.71 |
| 86 | Ethyl carbamate | 1.49 | 0.036 | 0.69 |
| 87 | 18-Oxooleate | 1.96 | 0.008 | 0.68 |
| 88 | 16-Oxohexadecanoic acid | 2.07 | 0.007 | 0.67 |
| 89 | Sterebin B | 1.49 | 0.018 | 0.59 |
| 90 | L-Prolyl-L-methionine | 1.84 | 0.002 | 0.59 |
| 91 | Prostaglandin K1 | 1.61 | 0.044 | 0.59 |
| 92 | Ethyl N-acetyl-L-tyrosinate | 1.44 | 0.048 | 0.58 |
| 93 | 3-Methoxybenzyl alcohol | 1.58 | 0.005 | 0.58 |
| 94 | Tangeraxanthin | 2.39 | 0.014 | 0.58 |
| 95 | D-glutamine | 1.33 | 0.039 | 0.56 |
| 96 | 1-Octadecyl Lysophosphatidic Acid | 1.75 | 0.012 | 0.55 |
| 97 | Dehydroabietic acid | 1.71 | 0.032 | 0.55 |
| 98 | Theophylline | 1.72 | 0.044 | 0.53 |
| 99 | 16-Hydroxyhexadecanoic acid | 2.27 | 0.004 | 0.52 |
| 100 | Benzoic acid | 2.10 | 0.004 | 0.52 |
| 101 | 4-Guanidinobutyrate | 2.17 | 0.001 | 0.51 |
| 102 | 4-Hydroxyretinoic acid | 2.23 | <0.001 | 0.51 |
| 103 | 16alpha-Hydroxyandrostenedione | 2.00 | 0.011 | 0.51 |
| 104 | 1-Erucoylglycerol-3-phosphate | 2.44 | 0.001 | 0.50 |
| 105 | Glycyltryptophan | 1.70 | 0.035 | 0.50 |
| 106 | Ribonolactone | 1.92 | 0.021 | 0.50 |
| 107 | 18-carboxy dinor Leukotriene B4 | 1.27 | 0.036 | 0.50 |
| 108 | Ala-Lys | 2.20 | 0.002 | 0.49 |
| 109 | Leu-Gly | 1.42 | 0.036 | 0.49 |
| 110 | Threonylproline | 1.99 | 0.047 | 0.48 |
| 111 | 17-Hydroxylinolenic acid | 1.82 | 0.021 | 0.47 |
| 112 | Indolepyruvate | 2.11 | 0.003 | 0.46 |
| 113 | Phe-Val | 1.77 | 0.021 | 0.46 |
| 114 | Phe-Glu | 1.76 | 0.030 | 0.46 |
| 115 | Docosanedioic acid | 1.32 | 0.043 | 0.43 |
| 116 | Homocysteine | 1.84 | 0.023 | 0.41 |
| 117 | Cysteinyl-Phenylalanine | 2.17 | 0.024 | 0.40 |
| 118 | N-Acetyl-D-glucosamine | 2.24 | 0.028 | 0.38 |
| 119 | Gluconasturtiin | 1.87 | 0.016 | 0.36 |
| 120 | Carnitine-2-methyl-C4 | 1.61 | 0.030 | 0.36 |
| 121 | Lys-Lys | 2.11 | <0.001 | 0.34 |
| 122 | Glutaminylalanine | 1.41 | 0.013 | 0.33 |
| 123 | Ser-Phe | 1.55 | 0.035 | 0.32 |
| 124 | Thiamine | 2.27 | 0.011 | 0.31 |
| 125 | Prostaglandin D2 Ethanolamide | 2.45 | 0.014 | 0.27 |
| 126 | Prolylhistidine | 1.24 | 0.014 | 0.24 |
| 127 | Glucaric acid | 1.51 | 0.028 | 0.23 |

**Table S5.** Differential metabolites in fecal samples between CD mice and HSD mice. Only features with VIP > 1, *p* < 0.05 and Fold Change > 2 or < 0.5 were listed.

| **No.** | **Metabolites** | **VIP** | *p* value | **Fold Change** |
| --- | --- | --- | --- | --- |
| 1 | Carnitine C5:0(3-Methylbutyrylcarnitine) | 1.83 | <0.001 | 197.910 |
| 2 | Lys-Asp | 2.36 | <0.001 | 112.652 |
| 3 | Lys-Met | 1.67 | <0.001 | 42.004 |
| 4 | Phe-Val | 1.78 | <0.001 | 37.615 |
| 5 | Serotonin | 1.70 | <0.001 | 35.408 |
| 6 | Trp-tyr | 1.80 | <0.001 | 31.949 |
| 7 | Arg-Thr | 1.06 | 0.011 | 26.459 |
| 8 | Arg-Ala | 1.70 | 0.001 | 22.541 |
| 9 | Tyr-Cys | 1.39 | <0.001 | 21.484 |
| 10 | Arg-Lys | 1.07 | 0.010 | 20.850 |
| 11 | Gly-Ile | 1.81 | <0.001 | 20.686 |
| 12 | Pro-ala | 1.72 | <0.001 | 19.017 |
| 13 | Hydroxyprogesterone | 1.62 | <0.001 | 17.261 |
| 14 | Glu Gln | 1.44 | <0.001 | 17.160 |
| 15 | Sphinganine 1-phosphate | 1.74 | <0.001 | 16.490 |
| 16 | Asn-Val | 1.49 | <0.001 | 13.758 |
| 17 | Asp-Lys | 1.50 | <0.001 | 13.300 |
| 18 | Serylarginine | 1.51 | <0.001 | 13.016 |
| 19 | N-Acetylserotonin | 1.11 | 0.014 | 12.828 |
| 20 | Octadecylamine | 1.52 | <0.001 | 12.711 |
| 21 | Lys-Ser | 1.56 | 0.002 | 10.554 |
| 22 | L-isoleucyl-L-asparagine | 1.52 | <0.001 | 9.828 |
| 23 | Phe-Phe | 1.49 | <0.001 | 9.572 |
| 24 | Ser-Gln | 1.02 | 0.015 | 8.886 |
| 25 | Glu-Ala | 1.52 | <0.001 | 8.589 |
| 26 | Tyrosylserine | 1.35 | <0.001 | 8.174 |
| 27 | 3-Dehydroteasterone | 1.50 | <0.001 | 8.171 |
| 28 | DL-Histidyl-DL-histidine | 1.23 | 0.002 | 8.137 |
| 29 | Pro-Air | 1.47 | <0.001 | 8.092 |
| 30 | Trp-His | 1.43 | <0.001 | 7.437 |
| 31 | Carnitine C20:1 | 1.57 | <0.001 | 7.403 |
| 32 | Glucaric acid | 1.92 | <0.001 | 6.894 |
| 33 | His-Arg | 1.43 | <0.001 | 6.821 |
| 34 | Pentedrone | 1.53 | <0.001 | 6.515 |
| 35 | Tryptamine | 1.42 | 0.001 | 6.161 |
| 36 | His-Phe | 2.10 | <0.001 | 5.959 |
| 37 | N-Acetylisoleucine | 1.53 | <0.001 | 5.856 |
| 38 | Tyramine | 1.69 | <0.001 | 5.553 |
| 39 | Arg-Trp | 1.32 | <0.001 | 5.143 |
| 40 | Cymiazole | 1.32 | 0.001 | 5.015 |
| 41 | LysoPE(0:0/14:0) | 1.44 | <0.001 | 4.843 |
| 42 | Propiomazine | 1.25 | 0.002 | 4.658 |
| 43 | L-Leucine | 1.47 | <0.001 | 4.620 |
| 44 | Arg-Ile | 1.29 | 0.003 | 4.408 |
| 45 | Glycolithocholic acid | 1.42 | <0.001 | 4.229 |
| 46 | Glu-Arg | 1.25 | 0.001 | 4.110 |
| 47 | Sphingosine | 1.44 | <0.001 | 4.022 |
| 48 | Ser-Trp | 1.08 | 0.001 | 3.915 |
| 49 | 19-Oxotestosterone | 1.21 | <0.001 | 3.886 |
| 50 | Gluconasturtiin | 1.52 | <0.001 | 3.864 |
| 51 | Cys-Tyr | 1.30 | 0.001 | 3.792 |
| 52 | Glu-His | 1.93 | <0.001 | 3.758 |
| 53 | Carnitine C18:1(Acylcarnitine C18:1) | 1.24 | 0.002 | 3.612 |
| 54 | Carnitine C20:1-OH | 1.02 | 0.001 | 3.460 |
| 55 | Tyrosyl-Valine | 1.30 | 0.005 | 3.372 |
| 56 | Sphinganine | 1.56 | <0.001 | 3.361 |
| 57 | Estrone | 1.28 | <0.001 | 3.316 |
| 58 | Betulonic acid | 1.69 | <0.001 | 3.280 |
| 59 | Formetanate | 1.23 | 0.002 | 3.176 |
| 60 | Carnitine C16:1 | 1.15 | <0.001 | 3.097 |
| 61 | L-Saccharopine | 1.24 | 0.009 | 2.892 |
| 62 | Tetradecylamine | 1.01 | 0.001 | 2.822 |
| 63 | L-Isoleucine | 1.15 | <0.001 | 2.784 |
| 64 | Arg-Arg | 1.32 | <0.001 | 2.770 |
| 65 | 1-Methyladenosine | 1.56 | 0.003 | 2.676 |
| 66 | Inosine | 1.20 | 0.005 | 2.558 |
| 67 | Carnitine C9:1-OH | 1.09 | 0.028 | 2.522 |
| 68 | Asn-Tyr | 1.20 | <0.001 | 2.343 |
| 69 | Eicosapentaenoic acid | 1.44 | <0.001 | 2.275 |
| 70 | His-Ile | 1.28 | 0.001 | 2.270 |
| 71 | Trp-Asn | 1.54 | <0.001 | 2.144 |
| 72 | Sphingosine 1-phosphate | 1.39 | <0.001 | 2.110 |
| 73 | Methylamine | 1.20 | 0.001 | 2.061 |
| 74 | N-Acetyl-L-phenylalanine | 1.22 | <0.001 | 0.481 |
| 75 | Glu-glu | 1.07 | 0.001 | 0.473 |
| 76 | Adlupone | 1.13 | <0.001 | 0.457 |
| 77 | Indoleacetic acid | 1.14 | 0.004 | 0.417 |
| 78 | Hexadecanedioic acid | 1.20 | <0.001 | 0.400 |
| 79 | Hexaethylene glycol | 1.20 | 0.001 | 0.397 |
| 80 | L-Prolyl-L-methionine | 1.19 | <0.001 | 0.378 |
| 81 | Glycine dehydrocholic acid | 1.09 | 0.006 | 0.375 |
| 82 | 1-Hydroxyisoquinoline | 1.24 | <0.001 | 0.356 |
| 83 | Retrocalamin | 1.31 | <0.001 | 0.342 |
| 84 | Ethylmethylacetic acid | 1.24 | <0.001 | 0.336 |
| 85 | Linoelaidic acid | 1.43 | <0.001 | 0.325 |
| 86 | L-Hexanoylcarnitine | 1.29 | <0.001 | 0.286 |
| 87 | 19-Hydroxytestosterone | 1.63 | <0.001 | 0.286 |
| 88 | Asn-Trp | 1.54 | <0.001 | 0.268 |
| 89 | Taurine | 1.77 | 0.001 | 0.266 |
| 90 | Indolepyruvate | 1.47 | 0.013 | 0.260 |
| 91 | 1-Methylguanosine | 1.54 | 0.005 | 0.258 |
| 92 | Hydroxyvalerenic Acid | 1.20 | 0.001 | 0.256 |
| 93 | Ile-Gln | 1.13 | <0.001 | 0.253 |
| 94 | D-piperidine acid | 1.50 | <0.001 | 0.245 |
| 95 | Farnesyl acetate | 1.39 | <0.001 | 0.242 |
| 96 | Hydroxypyruvic acid | 1.56 | <0.001 | 0.237 |
| 97 | Linoleoyl ethanolamide | 1.57 | <0.001 | 0.230 |
| 98 | 4-Oxoretinoic acid | 1.22 | <0.001 | 0.228 |
| 99 | Alpha-Linoleoylcholine | 1.42 | <0.001 | 0.206 |
| 100 | 4-Guanidinobutyric acid | 1.41 | <0.001 | 0.206 |
| 101 | Dodecanoic acid | 1.47 | <0.001 | 0.200 |
| 102 | L-Alanyl-L-alanine | 1.34 | <0.001 | 0.195 |
| 103 | 3-Hexenedioic acid | 1.48 | <0.001 | 0.194 |
| 104 | Diethyl adipate | 1.43 | <0.001 | 0.190 |
| 105 | Pro-Ser | 1.69 | <0.001 | 0.189 |
| 106 | alpha-Linolenic acid | 1.67 | <0.001 | 0.187 |
| 107 | Kynuramine | 1.35 | <0.001 | 0.185 |
| 108 | Val-Gln | 1.30 | <0.001 | 0.178 |
| 109 | Ethyl alpha-linolenate | 1.46 | <0.001 | 0.167 |
| 110 | D-Ornithine | 1.45 | <0.001 | 0.158 |
| 111 | N-Acetyl-leucyl-leucine | 1.48 | <0.001 | 0.157 |
| 112 | LysoPC(22:2(13Z,16Z) | 1.39 | <0.001 | 0.155 |
| 113 | 7,8-diaminopelargonate | 1.61 | <0.001 | 0.154 |
| 114 | Butyl butyryllactate | 1.19 | <0.001 | 0.145 |
| 115 | Oxoglutaric acid | 1.96 | 0.003 | 0.126 |
| 116 | Toluene | 1.69 | <0.001 | 0.122 |
| 117 | leukotriene D4 | 1.20 | 0.002 | 0.120 |
| 118 | Leu-Ile | 1.63 | <0.001 | 0.115 |
| 119 | Stearidonic acid | 1.58 | <0.001 | 0.112 |
| 120 | Tyrosylthreonine | 1.19 | <0.001 | 0.111 |
| 121 | Diethyl succinate | 1.59 | <0.001 | 0.106 |
| 122 | Tyrosol | 1.47 | <0.001 | 0.105 |
| 123 | Adenosine 5'-triphosphate（ATP） | 1.22 | <0.001 | 0.101 |
| 124 | Diethylpropion | 1.55 | <0.001 | 0.100 |
| 125 | Pelargonic acid | 1.51 | <0.001 | 0.096 |
| 126 | L-Phenylalaninol | 1.62 | <0.001 | 0.089 |
| 127 | FAA(18:1) | 1.48 | <0.001 | 0.087 |
| 128 | FAA(17:1) | 1.49 | <0.001 | 0.085 |
| 129 | Cholesterol | 1.79 | <0.001 | 0.058 |
| 130 | Tetradecanedioic acid | 1.66 | <0.001 | 0.020 |

**Table S6.** Q value of tryptophan metabolites directly combined with three concentrations of FOLFOX.

| **No.** | **Tryptophan metabolite** | | **F1** | **F2** | **F3** |
| --- | --- | --- | --- | --- | --- |
| 1 | | TP | 1.11 | 1.09 | 1.11 |
| 2 | | TM | 1.21 | 1.12 | 1.17 |
| 3 | | IN | 1.06 | 1.02 | 1.09 |
| 4 | | SK | 0.92 | 0.88 | 0.91 |
| 5 | | ILA | 1.02 | 0.98 | 1.00 |
| 6 | | IPA | 1.03 | 0.96 | 1.04 |
| 7 | | I3A | 0.90 | 0.86 | 0.85 |
| 8 | | 3HK | 0.90 | 0.87 | 0.86 |
| 9 | | IAA | 1.03 | 0.88 | 0.96 |

Q < 0.85 is antagonistic, 0.85 ≤ Q < 1.15 is additive, and Q ≥ 1.15 is synergistic. Nine tryptophan metabolites: TP: Tryptophan, 50 μM; TM: Tryptamine, 5 μM; IN: Indole, 500 μM; SK: Skatole, 100 μM; ILA: Indole-3-Lactic Acid, 800 μM; IPA: 3-Indole propionic acid, 500 μM; I3A: Indole-3-carboxaldehyde, 100 μM; 3HK: Kynurenine, 500 μM; IAA: 3-Indoleacetic acid, 1000 μM.
